# Cyanoglycosides isolated from *Moringa oleifera* seeds inhibited PFKFB3/TGF-β1/smads pathway to alleviate diabetic nephropathy through driving metabolic reprogramming

**DOI:** 10.1080/13880209.2025.2607563

**Published:** 2025-12-29

**Authors:** Chengyu Ge, Zhihua Shi, Jia He, Xu Feng, Kaiqi Shang, Xiaolin Liao, Yufeng Liu, Yueping Jiang, Shao Liu

**Affiliations:** a Department of Pharmacy, Xiangya Hospital, Central South University, Changsha, Hunan, China; b National Clinical Research Center for Geriatric Disorders, Xiangya Hospital, Central South University, Changsha, Hunan, China; c Department of Clinical Pharmacy, Hunan University of Medicine General Hospital, Huaihua, Hunan, China; d College of Pharmacy, Jining Medical University, Rizhao, Shandong, China; e Hunan Provincial Key Laboratory of the Research and Development of Novel Pharmaceutical Preparations, “The 14th Five-Year Plan” Application Characteristic Discipline of Hunan Province (Pharmaceutical Science), College of Pharmacy, Changsha Medical University, Changsha, Hunan, China

**Keywords:** *Moringa oleifera* seeds, cyanoglycosides, structure elucidation, diabetic nephropathy, metabolic reprogramming

## Abstract

**Context:**

Diabetic nephropathy (DN) is a major complication of diabetes. *Moringa oleifera* seeds are recognized as a source of bioactive compounds with potential health benefits, prompting investigation into their specific components and effects on DN.

**Objective:**

This study aimed to isolate bioactive compounds from *M. oleifera* seeds and evaluate their renoprotective effects and underlying mechanisms of action against high-glucose-induced diabetic nephropathy.

**Materials and methods:**

Four cyanoglycosides and one cyanoaglycone were isolated from *M. oleifera* seeds using chromatographic techniques. The renoprotective effects of these compounds were then evaluated using an *in vitro* model of high-glucose-induced diabetic nephropathy in HBZY-1 mesangial cells. Mechanistic studies further investigated the compounds’ effects on oxidative stress, inflammation, mitochondrial function, expression of the glycolysis-related protein PFKFB3, and the TGF-β1/Smad signaling pathway.

**Results:**

Two previously undescribed cyanoglycosides were isolated alongside three known compounds. All five compounds demonstrated significant renoprotective effects in the high-glucose-induced HBZY-1 cell model. Mechanistically, these effects were achieved by suppressing oxidative stress and inflammation, protecting mitochondrial function, modulating the expression of the glycolysis-related protein PFKFB3, and inhibiting the TGF-β1/Smad signaling pathway, collectively contributing to beneficial metabolic reprogramming.

**Conclusions:**

This study isolated two novel cyanoglycosides from *M. oleifera* seeds. These compounds, alongside known ones, protect against high-glucose-induced renal injury. Their renoprotection involves metabolic reprogramming *via* suppressing oxidative stress/inflammation, preserving mitochondrial function, modulating PFKFB3, and inhibiting TGF-β1/Smad signaling. These findings offer insights for utilizing *M. oleifera* seeds and suggest these cyanoglycosides as potential diabetic nephropathy therapeutics.

## Introduction

Diabetic nephropathy (DN), a serious complication of diabetes (Zhang et al. 2024), is the primary cause of end-stage renal disease worldwide (Patidar et al. 2024). Its defining pathological feature is chronic kidney injury resulting from disrupted glucose metabolism (Chen et al. 2024). DN progression manifests as proteinuria, which ultimately leads to renal interstitial fibrosis and glomerulosclerosis. Current first-line therapies predominantly target the renin-angiotensin system through angiotensin-converting enzyme inhibitors or angiotensin II type 1 receptor blockers (Li et al. 2020). However, these interventions fail to fully mitigate the residual risk of DN progression, resulting in substantial clinical burden. Critically, no existing pharmacotherapies directly address the underlying pathogenic mechanisms or effectively halt the disease progression. This therapeutic gap underscores the urgent need for novel strategies targeting DN progression in diabetes research.

Metabolic reprogramming, a key pathogenic mechanism in diabetic nephropathy (DN), is characterized by dysfunctional mitochondrial biosynthesis, enhanced glycolysis, and dysregulated lipid and amino acid metabolism. This process involves adaptive alterations in cellular metabolic pathways that sustain aberrant cell growth and proliferation (Fan et al. 2024). Concurrently, metabolic derangements drive DN progression through oxidative stress, inflammatory damage, and dysregulation of autophagy/apoptosis, ultimately culminating in renal fibrosis (Fan et al. 2024). Notably, mitochondrial dysfunction and impaired glycolysis constitute critical pathomechanisms underlying podocyte injury in diabetic kidney disease (Zhang et al. 2021; Kanasaki, 2023). The TGF-β1 signaling pathway has been identified as a central mediator of DN pathogenesis. It also promotes extracellular matrix (ECM) protein deposition, a hallmark of glomerulosclerosis (Hathaway et al. 2015; Liu et al. 2023). Mechanistically, TGF-β binding to its cognate receptor activates downstream Smad2/3 signaling, which is pivotal for modulating glomerulosclerosis progression (Liu et al. 2023). It is worth mentioning that glycolysis dysfunction is an important pathogenesis of podocyte injury in diabetic kidney disease (Zhang et al. 2021). Fructose-2,6-biphosphatase 3 (PFKFB3) is a bifunctional enzyme that plays crucial roles in glycolysis, cell proliferation, survival, and adhesion (Huang et al. 2023). PFKFB3 expression is positively correlated with the severity of renal fibrosis severity (Wang et al. 2024b). However, the functional interplay between PFKFB3-mediated glycolytic regulation and TGF-β/Smad2/3 signaling in ameliorating podocyte injury remains unexplored.

Natural medicines, particularly traditional Chinese medicines, serve as vital sources of bioactive lead compounds for drug discovery and have diverse pharmacological activities (Bai et al. 2023; Malczak and Gajda, 2023; Qu et al. 2023). *Moringa oleifera* Lam. (family Moringaceae), native to India and commonly known as the "Tree of Life" (Dantas et al. 2024), is widely distributed in tropical and subtropical regions. This species is valued both as a nutritional resource and traditional remedy (Leone et al. 2016; Randriamboavonjy et al. 2017). *Moringa oleifera* is not only valuable as a food source (Cirlini et al. 2022), but its leaves, seeds, stems, and roots also have medicinal properties for the treatment of diabetes, paralysis, helminthiasis, sores, and skin infections (Sha et al. 2020). The *M. oleifera* seeds are particularly notable for their high oil content and nutritional profile (Saa et al. 2019). Phytochemical analyses have identified diverse constituents of seeds, including flavonoids, carbamates, glucosinolates, phenols, steroids, carotenoids, amino acids, sugars, and essential minerals (Chen et al. 2019; Dzuvor et al. 2022; Peng et al. 2024). *M. oleifera* seeds exhibit a variety of pharmacological activities, owing to their diverse chemical constituents. These chemical constituents or the extracts of *M. oleifera* seeds have been discovered to possess antibacterial activity (Salem et al. 2021), antiviral properties (Xiong et al. 2021), blood pressure and blood sugar-lowering effects (Wang et al. 2017), anti-inflammatory activity (Sailaja et al. 2021; Xiong et al. 2022), gastrointestinal spasm relief (Sadraei et al. 2015), and anti-obesity activity (Elarabany et al. 2022) among others (Liu et al. 2022a).

Cyanogenic glycosides, characterized by a hydroxyl group at the α-position of the cyanohydrin moiety, are distributed across approximately 3,000 plant species. To date, more than 100 structurally distinct cyanogenic glycosides have been identified, with diversity arising from variations in precursor amino acids and subsequent enzymatic modifications (Sánchez-Pérez and Neilson, 2024). In contrast, non-cyanogenic glycosides have comparatively low natural prevalence (Wang et al. 2011). These non-cyanogenic glycosides demonstrate multiple bioactivities, including antihypertensive activity (Faizi et al. 1994), enhanced antibiotic activity against Gram-positive and Gram-negative bacteria (Wang et al. 2011), antioxidant activity (Wang et al. 2021), and anti-diabetic activity (Bao et al. 2020).

Although our prior investigations have established the anti-diabetic nephropathy potential of *Moringa oleifera* seed extracts (Wen et al. 2022), the therapeutic effects of their specific chemical constituents remain uncharacterized. To date, only a limited number of cyanoglycosides have been identified in *M. oleifera* seeds (Faizi et al. 1994; Liu et al. 2022a), and a systematic evaluation of their renoprotective efficacy in diabetic nephropathy remains lacking. As part of an ongoing research program to explore the chemical diversity and bioactivity profiles of traditional medicinal plants (Jiang et al. 2022; Wen et al. 2022; Liu et al. 2022a; Jiang et al. 2023; Wang et al. 2024c), we systematically investigated the bioactive components of *M. oleifera* seeds and their pharmacological relevance.

**Figure 1.**
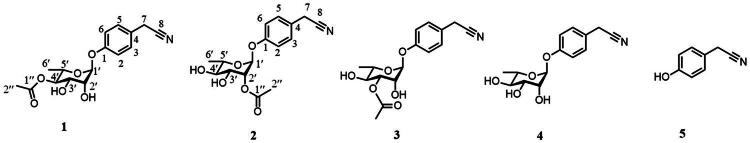
Structures of compounds **1**–**5**.

The isolation, structural elucidation, antidiabetic nephropathy activity, and mechanism involving metabolic reprogramming investigation of four cyanoglycosides and one cyanoaglycone, including two undescribed cyanoglycosides, are described herein (Figure 1).

## Materials and methods

### General experimental procures

^1^H and ^13^C NMR spectra were obtained at 600 MHz and 150 MHz using a Bruker 600 MHz spectrometer (Bruker, Billerica, MA, USA), with the tetramethylsilane (TMS) peak used as a reference. The HR-ESI-MS data were measured using an Agilent Q-TOF spectrometer (Agilent Technologies). Column chromatography (CC) was performed using silica gel (200–300 mesh; Qingdao Marine Chemical, Qingdao, China), Sephadex LH-20 (Sigma-Aldrich, St. Louis, MO, USA), and MCI gel (CHP20P) (Mitsubishi Chemical, Tokyo, Japan). HPLC separation was performed using an Agilent 1260 system, an Agilent 1260 pump, and an Agilent 1260 wavelength absorbance detector with an Agilent (250 × 9.4 mm) semi-preparative column packed with C18 (5 μm) (Agilent Technologies) and a YMC (250 × 10 mm) semi-preparative column coated with 5 μm phenyl silica gel (YMC, Kyoto, Japan). TLC separations were performed on pre-coated silica gel GF254 plates (Qingdao Marine Chemical). spots were visualized under UV light (254 or 356 nm) or by spraying with 10% H_2_SO_4_ in 90% EtOH, followed by heating.

### Plant material

*M. oleifera* seeds were collected from Kunming City, Yunnan Province, China (latitude: 24°88′N; Longitude:102° 88′ E), in September 2019 (dry season). This study complies with local legislation. This plant is not protected, and the local government permits harvesting. The plant identity was verified by Professor Shao Liu (Xiangya Hospital, Central South University, Changsha, 410008, China). A voucher specimen (ID 2,019,001) was deposited in the authors’ laboratory at the Department of Pharmacy, Xiangya Hospital, Central South University, Changsha, 410008, China.

### Extraction and isolation

The extraction and rough partitioning processes have been reported previously (Jiang et al. 2022; 2023; Wang et al. 2024c). To further investigate the bioactive compounds present in *M. oleifera* seeds, *M. oleifera* seed ethanol extracts were isolated and purified. The ethanol extracts (413 g) were subjected to CC over silica gel, with elution by a gradient of increasing MeOH concentration (0–100%) in CH_2_Cl_2_ to yield fractions LMZC-1–LMZC-12 based on TLC analysis. Fraction LMZC-1 (∼21.1 g) was separated by column chromatography (CC) over MCI gel CHP 20P (5 L) with successive elution using EtOH–H_2_O (2:8) (20 L), EtOH–H_2_O (4:6) (20 L), EtOH–H_2_O (6:4) (20 L), and EtOH (20 L) to obtain fractions LMZC-1–1–LMZC-1–4. Fraction LMZC-1–2 (∼150 mg) was purified by RP HPLC (C18 column, 2.0 mL/min) using 20% MeCN in H_2_O containing 1 ‰ formic acid (v/v) as the mobile phase to yield fractions LMZC-1-2-1–LMZC-1-2-8. Fraction LMZC-1-2-5 (∼30 mg) was purified by RP HPLC (Phenyl column, 2.0 mL/min), using 30% MeOH in H_2_O containing 1‰formic acid (v/v) as the mobile phase to yield **5** (8.0 mg, *t*_R_ = 23.6 min). Fraction LMZC-3 (∼1.5 g) was separated by RP HPLC (C18 column, 2.0 mL/min) using 28% MeCN in H_2_O containing 1‰ formic acid (v/v) as the mobile phase to yield fractions LMZC-3-1–LMZC-3-7. Fraction LMZC-3-1 (∼100 mg) was further purified by RP HPLC (Phenyl column, 2.0 mL/min) using 23% MeCN in H_2_O containing 1‰ formic acid (v/v) as the mobile phase, to yield **3** (3.0 mg, *t*_R_ = 27.9 min). Fraction LMZC-3-2 (∼50 mg) was further purified by RP HPLC (Phenyl column, 2.0 mL/min) using 22% MeCN in H_2_O containing 1‰ formic acid (v/v) as the mobile phase, to yield **2** (∼1.0 mg, *t*_R_ = 36.0 min). Fraction LMZC-3-3 (∼80 mg) was further purified by RP HPLC (Phenyl column, 2.0 mL/min) using 50% MeOH in H_2_O containing 1‰ formic acid (v/v) as the mobile phase, to yield **1** (8.0 mg, *t*_R_ = 23.4 min). Fraction LMZC-4 (∼0.5 g) was separated by RP HPLC (C18 column, 2.0 mL/min) using 20% MeCN in H_2_O containing 1‰ formic acid (v/v) as the mobile phase to yield fractions LMZC-4-1–LMZC-4-3. Fraction LMZC-4-1 (∼60 mg) was further purified by RP HPLC (Phenyl column, 2.0 mL/min) using 15% MeCN in H_2_O containing 1‰ formic acid (v/v) as the mobile phase, to yield **4** (5.0 mg, *t*_R_ = 23.9 min).

### Biological activity and mechanism investigation assay

#### Cell culture and model establishment

Glomerular mesangial cells are key effector cells in DN development, and their abnormal proliferation, inflammatory response, and extracellular matrix deposition in a high-glucose environment are the typical pathological features of DN. Rat glomerular mesangial cells (HBZY-1), a rat-derived mesangial cell line (CVCL_7213), directly mimic the pathological behavior of mesangial cells in DN. HBZY-1 cells were purchased from Boster Biological Technology (Catalogue number: CX0128, Wuhan, China). The cells were maintained in Dulbecco’s modified Eagle’s medium (DMEM) supplemented with 10% fetal bovine serum (FBS), 100 μg/mL streptomycin sulfate, and 100 U/mL penicillin G sodium salt in a humidified atmosphere with 5% CO_2_ at 37 °C. Next, 20 μL DMEM was added to the blank and control wells. HBZY-1 cells were seeded into 96-well plates at a density of 5 × 10^3^ cells per well, followed by exposure to a final glucose concentration of 30 mM glucose (HG) for 24 h to simulate the high-glucose microenvironment of diabetic nephropathy (Wang et al. 2024a).

#### HBZY-1 cells proliferation assay

HBZY-1 Cell proliferation was detected using the Cell Counting Kit-8 (CCK-8) assay. HBZY-1 cells in the logarithmic growth phase were seeded at a density of 5 × 10^3^ cells/well in 96-well plates and incubated at 37 °C for 24 h in a humidified atmosphere containing 5% CO_2_. Cells were treated with normal glucose or high glucose (30 mM) in the absence or presence of compounds **1**, **3**, **4**, and **5** with different concentrations (10, 20, 50, and 100 μM) were added to each well, respectively. This was followed by incubation at 37 °C with 5% CO_2_ for 24 h. The medium was removed, and cells were incubated with Cell Counting Kit-8 reagent (100 μL, CCK-8, Dojindo, Kyushu Island, Japan) at 37 °C for 24 h in a CO_2_ incubator, respectively. Absorbance was measured at 450 nm in each well using a Bio-Tek microplate reader (Huisong Technology, Guangdong Province, China).

#### The mechanism investigation of compounds alleviates diabetic kidney damage

##### Measurement of ROS production

ROS production in HG-treated HBZY-1 cells treated with compounds **1**, **3**, **4**, and **5** was analyzed by immunofluorescence analysis. Briefly, HBZY-1 cells were plated in 12-well plates and exposed to high concentrations. The cells were treated with compounds **1**, **3**, **4**, or **5** (20 μM) for 24 h. An ROS detection kit (Beyotime, Shanghai, China) was used to determine ROS levels in HBZY-1 cells. The procedure involved adding 1 μM 2′,7′-Dichlorodihydrofluorescein diacetate (DCFH-DA) probe diluted in serum-free DMEM, followed by incubation at 37 °C with 5% CO_2_ for 30 min. Unbound probes were removed by three washes with prewarmed PBS. The fluorescence was visualized using an inverted microscope (N2Ti2-A; Zeiss, Germany). Three replicate wells per group were analyzed using random field selection per well. ImageJ software was used to quantify the average fluorescence intensity, and the ROS generation rates were normalized to those of the normoglycemic control group.

#### Measurement of mitochondrial membrane potential

High-glucose-induced HBZY-1 cells were plated in 12-well plates and treated with compounds **1**, **3**, **4**, or **5** (20 μM) for 24 h. Mitochondrial membrane potential (MMP) changes were assessed using a JC-1 assay kit (Beyotime, cat. no. C2006, China) following the manufacturer’s protocol. JC-1 working solution was added at the designated concentration and incubated at 37 °C for 30 min in the dark. After incubation, the cells were washed thrice with JC-1 staining buffer and visualized under an inverted fluorescence microscope (N2Ti2-A, Zeiss, Germany). MMP loss was quantified as the ratio of red fluorescence (aggregates) to green fluorescence (monomer) ratio. Three replicate wells per group were analyzed using random field selection per well. The H1-UL (red)/H1-UR (green) ratio was calculated to reflect mitochondrial membrane potential dynamics.

#### Measurement of inflammation factors

HBZY-1 cells were seeded in 12-well plates, induced with high glucose for 24 h, and subsequently treated with 20 μM compounds **1**, **3**, **4**, or **5** for 24 h. Following centrifugation at 4000 g for 20 min, cell supernatants were collected and stored at −20 °C. Cytokine concentrations of IL-1β (RX302869R), IL-6 (RX302856R), and TNF-α (RX302058R) were quantified using ELISA kits (Quanzhou Ruixin Biological Technology, Fujian, China) according to the manufacturer’s protocol. Optical density (OD) at 450 nm was measured using a BioTek microplate reader (Huisong Technology, Guangdong Province, China).

#### Western blotting

HBZY-1 cells were incubated with the test compounds under high glucose conditions. Cells were collected and lysed in a total lysis buffer containing protease inhibitors (100 μL). After incubation at −20 °C for 20 min, lysates were centrifuged at 4 °C (15,000 rpm, 15 min). The protein concentrations were quantified using a bicinchoninic acid assay kit (Beyotime, Shanghai, China). Proteins were separated by 8% SDS-PAGE and electrophoretically transferred onto PVDF membranes (Merck Millipore, Burlington, MA, USA) for 1.5 h (80 V). Membranes were blocked with 5% nonfat milk and incubated overnight at 4 °C with primary antibodies against PFKFB3 (A22317, ABclonal), TGF-β (AF1027, Affinity), Smad2 (12570-1-AP, Proteintech), Smad3 (12570-1-AP, Proteintech), and β-actin (AF7018, Affinity). After washing, the membranes were incubated with HRP-conjugated secondary antibodies (1:5000; Zhongshan Jinqiao Biotechnology, Beijing, China) at room temperature for 1 h. The detailed information of primary and secondary antibodies used in this study are listed in Table S1 (Please see the supporting information). Protein bands were visualized using an enhanced chemiluminescence kit (Beyotime, Shanghai, China) and analyzed using ImageJ software.

### Statistical analysis

All data are expressed as mean ± SD (standard deviation). One-way analysis of variance (ANOVA) was performed using GraphPad Prism (GraphPad Software, Inc., USA). All the data are representative of at least three independent experiments.

## Results and discussion

### Structural elucidation

Compound **1** was obtained as a yellowish amorphous powder of [*α*]^25^_D_ −8.2 (*c* = 0.0002, MeOH). Its molecular formula, C_16_H_19_NO_6_, was established by HR-ESIMS at *m/z* 344.1111[M + Na]^+^ combined with NMR spectral data (Table 1) and indicated eight degrees of unsaturation. The IR spectrum confirmed the presence of hydroxyl groups (3442 cm^−1^), cyano group (2248 cm^−1^), carbonyl group (1738 cm^−1^), and aromatic ring (1607,1508 cm^−1^) functionalities. Its 1D-NMR spectra (Table 1) showed one 1,4-disubstituted aromatic ring [*δ*_H_ 7.30 (2H, d, *J* = 7.8 Hz, H-3,5), 7.09 (2H, d, *J* = 7.8 Hz, H-2,6); *δ*_C_ 157.3 (C-1), 130.4 (C-3,5), 126.1 (C-4), 118.0 (C-2,6)], one rhamnopyranosyl moiety [*δ*_H_ 5.46 (1H, brs, H-1′), 5.01(1H, t, *J* = 9.6 Hz, H-4′), 4.03 (1H, brs, H-2′), 3.99 (1H, dd, *J* = 9.6, 3.0 Hz, H-3′), 3.76 (1H, dq, *J* = 9.6, 6.0 Hz, H-5′), 1.10 (3H, d, *J* = 6.0 Hz, H-6′); *δ*_C_ 99.7 (C-1′), 75.3 (C-4′), 72.0 (C-2′), 70.2 (C-3′), 68.6 (C-5′), 17.9 (C-6′)], one carbonyl group [*δ*c 172.5 (C-1′′)], one cyano carbon [*δ*c 119.8 (C-8)], one methyl [*δ*_H_ 2.09 (3H, s, H-2′′); *δ*_C_ 21.0 (C-2′′)], and one aliphatic methylene [*δ*_H_ 3.83 (2H, s, H-7); *δ*_C_ 22.8 (C-7)].

**Table 1.** 1H And 13 C NMR spectral data for compounds **1**–**2** (*δ* in ppm)^a^.

No.	1		2	
	δ_H_	δ_C_	δ_H_	δ_C_
1		157.3		157.2
2	7.09 (d, *J* = 7.8 Hz)	118.0	7.08 (d, *J* = 7.8 Hz)	118.1
3	7.30 (d, *J* = 7.8 Hz)	130.4	7.30 (d, *J* = 7.8 Hz)	130.5
4		126.1		126.0
5	7.30 (d, *J* = 7.8 Hz)	130.4	7.30 (d, *J* = 7.8 Hz)	130.5
6	7.09 (d, *J* = 7.9 Hz)	118.0	7.08 (d, *J* = 8.4 Hz)	118.1
7	3.83 (s)	22.8	3.84 (s)	22.8
8		119.8		120.8
1′	5.46 (brs)	99.7	5.46 (brs)	99.7
2′	4.03 (brs)	72.0	5.18 (d, *J* = 1.8 Hz)	75.5
3′	3.99 (dd, *J* = 9.6, 3.0 Hz)	70.2	4.01 (dd, *J* = 9.6, 3.6 Hz)	70.8
4′	5.01 (t, *J* = 9.6 Hz)	75.3	3.42 (t, *J* = 9.6 Hz)	73.7
5′	3.76 (dq, *J* = 9.6, 6.0 Hz)	68.6	3.66 (m)	69.8
6′	1.10 (d, *J* = 6.0 Hz)	17.9	1.23 (d, *J* = 6.0 Hz)	18.0
1ʺ		172.5		172.9
2ʺ	2.09 (s)	21.0	2.13 (s)	21.0

^a^ NMR data(*δ*) were measured in MeOH-*d*_4_ at 600 MHz for ^1^H NMR and at 150 MHz for ^13^C NMR.

Assignments were made by a combination of 1D and 2D NMR experiments.

**Figure 2.**
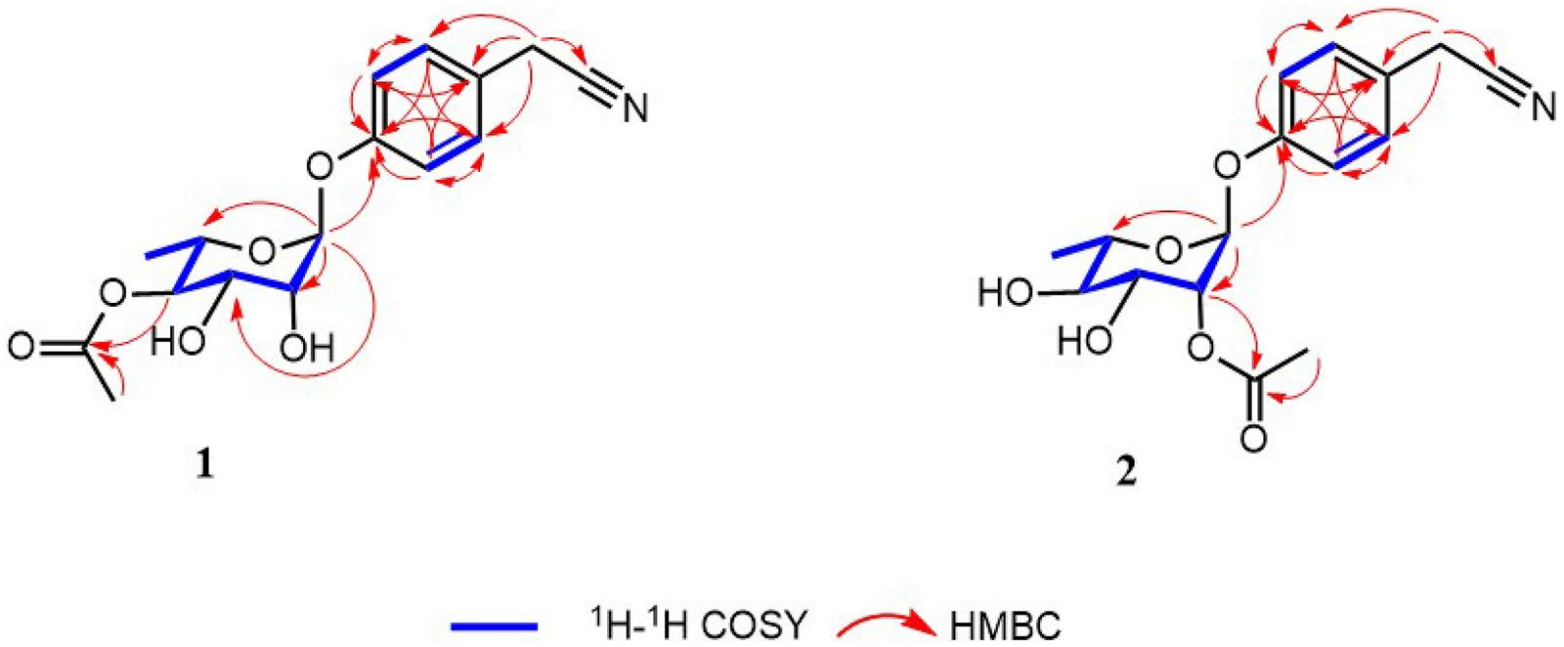
Key 1 H-1 H COSY and HMBC correlations of compounds **1**–**2**.

The DEPT, HSQC, and ^1^H–^1^H COSY spectra of **1** provided unambiguous assignments of proton and carbon signals in the NMR spectra. In the HMBC spectrum (Figure 2), the key correlations between H-7/C-3, C-4, C-5, and C-8; H-3,5/C-1, C-2, C-6, and C-7; and H-2,6/C-1, C-3, C-4, and C-5 establish the planar structure of *p*-phenylacetonitrile. The key correlations in the HMBC spectrum, H-1′/C-1, C-2′, C-3′, C-5′, and H-4′/C-1′′, in combination with the C-1 chemical shift (*δ*_C_ 157.3) and C-4′ chemical shift (*δ*c 75.3), demonstrated that one 4′-O-acetyl-rhamnopyranosyloxyl moiety was located at C-1 of *p*-phenylacetonitrile. The *α*-rhamnopyranosyl moiety was determined to be *α*-L-rhamnopyranosyl moiety by using a previously reported method (Liu et al. 2022b). Thus, the structure of **1** was determined to be (–)-1-(4′-O-acetyl-rhamnopyranosyloxyl)phenylacetonitrile and was named as cyanoleifera A.

Compound **2** was obtained as a yellowish powder with C_16_H_19_NO_6_ (eight degrees of unsaturation) using the HR-ESI-MS data (*m/z* 344.1110 [M + Na]^+^, calculated for C_16_H_19_NO_6_Na, 344.1105). The IR spectrum of compound **2** was similar to that of compound **1**, confirming the presence of hydroxyl groups (3381 cm^−1^), cyano groups (2244 cm^−1^), and aromatic ring (1607,1504 cm^−1^) functionalities. A comparison of the NMR spectral data of **2** and **1** indicated that the O-acetyl moiety was located at C-4′ in **1,** instead of the O-acetyl moiety located at C-2′ in **2** (Table 1). UV, IR, and NMR data analyses confirmed that **2** was an analog of **1**. In particular, the HMBC correlations from H-2′ to C-1′′, in combination with their chemical shifts, demonstrated that the O-acetyl moiety was located at the C-2′ position of **2** (Figure 2). The *α*-rhamnopyranosyl moiety was determined to be *α*-L-rhamnopyranosyl moiety by using a previously reported method (Liu et al. 2022b). Therefore, compound **2** was determined to be (–)-1-(2′-O-acetyl-rhamnopyranosyloxyl) phenylacetonitrile and was named as cyanoleifera B.

Cyanoleiferas A and B were identified as previously unreported compounds through an extensive search of SciFinder database. The known compounds were identified by comparing their spectroscopic data with reported data for niazirinin (**3**)(Atolani et al. 2020), niazirin (**4**) (Faizi et al. 1994), and p-hydroxyphenylacetonitrile (**5**) (Chen et al. 2014) (Figure 1).

### Physicochemical properties of compounds 1 and 2

#### Cyanoleifera A (1)

Yellowish amorphous powder; [*α*]^25^_D_ −8.2 (*c* = 0.0002, MeOH). UV (MeOH) *λ*_max_ 202, 222, 272 nm; IR *ν*_max_ 3442, 2978, 2933, 2850, 2248, 1738, 1607, 1508, 1373, 1229, 1120, 1018, 976, 822 cm^−1^; ^1^H NMR (CD_3_OD, 600 MHz) data, see Table 1; ^13^C NMR (CD_3_OD, 150 MHz) data, see Table 1; (+)-HRESIMS *m/z* 344.1111 [M + Na]^+^ (calcd for C_16_H_20_NO_6_Na, 344.1105).

#### Cyanoleifera B (2)

Yellowish amorphous powder; [*α*]^25^_D_ −20 (*c* = 0.00378, MeOH). UV (MeOH) *λ*_max_ 202, 222, 272 nm; IR *ν*_max_ 3381, 2923, 2846, 2244, 1607, 1504, 1373, 1232, 1123, 1059, 979, 829 cm^−1^; ^1^H NMR (CD_3_OD, 600 MHz) data, see Table 1; ^13^C NMR (CD_3_OD, 150 MHz) data, see Table 1; (+)-HRESIMS *m/z* 344.1110 [M + Na]^+^ (calcd for C_16_H_20_NO_6_Na, 344.1105).

### Biological evaluation and mechanism investigation

#### The effect of cyanoglycosides on the proliferation of HBZY-1 cells induced by high-glucose

**Figure 3.**
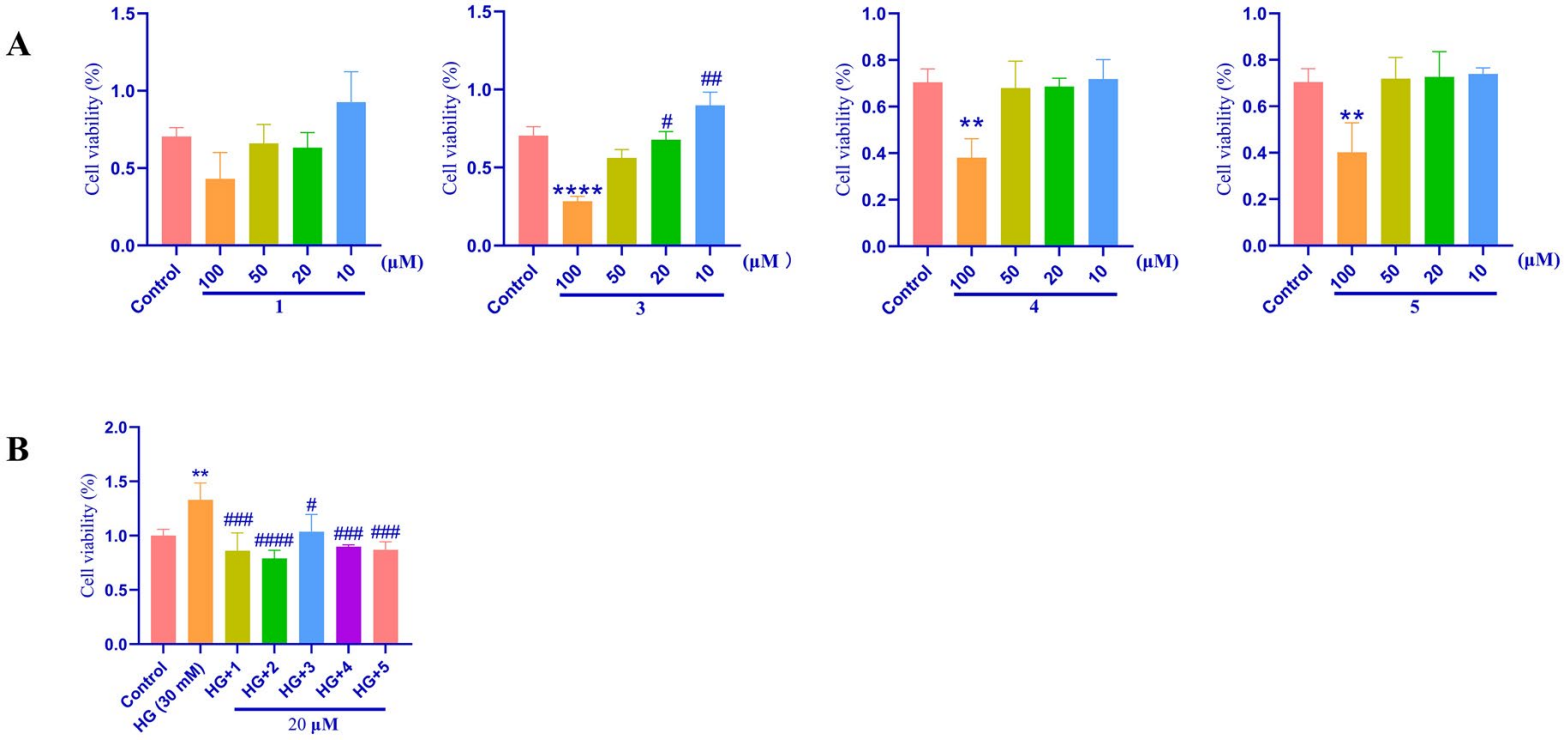
Effects of compounds **1**, **3**, **4**, **5** on HBZY-1 cell proliferation. (A) HBZY-1 cells were cultured with various concentrations of compounds **1**, **3**, **4**, **5** under normal glucose conditions. Note: No significant difference was observed between various concentrations of compound 1 and the control group. (B) HBZY-1 cells were incubated with high glucose (HG) medium to induce proliferation and then cultured with compounds **1**, **3**, **4**, **5** at various concentrations. Control group was no treated with high glucose and compounds. HG group was model group that was treated with high glucose (30 mM). HG + **1/2/3/4/5** groups were treated high glucose and corresponding compound. Data are presented as means ± SEMs (*n* = 3). **p < 0.01, vs. The control group, ***p < 0.001, vs. The control group, ****p < 0.0001, vs. The control group, #*p* < 0.05, vs. The HG group, ##*p* < 0.01, vs. The HG group.

Exposure to high glucose induced abnormal HBZY-1 cell proliferation, as evidenced by a significant increase in cell viability in the model group compared to the normal control, confirming the successful establishment of the model. Due to limited compound availability, only compounds **1**, **3**, **4**, and **5** were selected for *in vitro* evaluation of their protective effects against diabetic nephropathy. Initial dose-response experiments (0, 10, 20, and 50 μM) revealed no significant effect of these compounds on HBZY-1 cell proliferation (Figure 3A). Thus, four compounds treated with 20 μM to conduct follow-up anti-diabetic nephropathy effects using a high glucose-induced DN model in HBZY-1 cells. As shown in Figure 3B, the cell viability of the model group (high glucose-treated with HBZY-1) was significantly higher than that of the control group. However, compared to the high glucose-induced group, all compounds (**1**, **3**, **4**, and **5**) (20 μM) significantly reduced the viability of HBZY-1 cells. These results suggest that high glucose directly stimulates the proliferation of HBZY-1 cells and that the four cyanoglycosides have a significant inhibitory effect on HG-induced HBZY-1 cell proliferation.

#### Cyanoglycosides protect HBZY-1 cells against high-glucose induced reactive oxygen and inflammation

**Figure 4.**
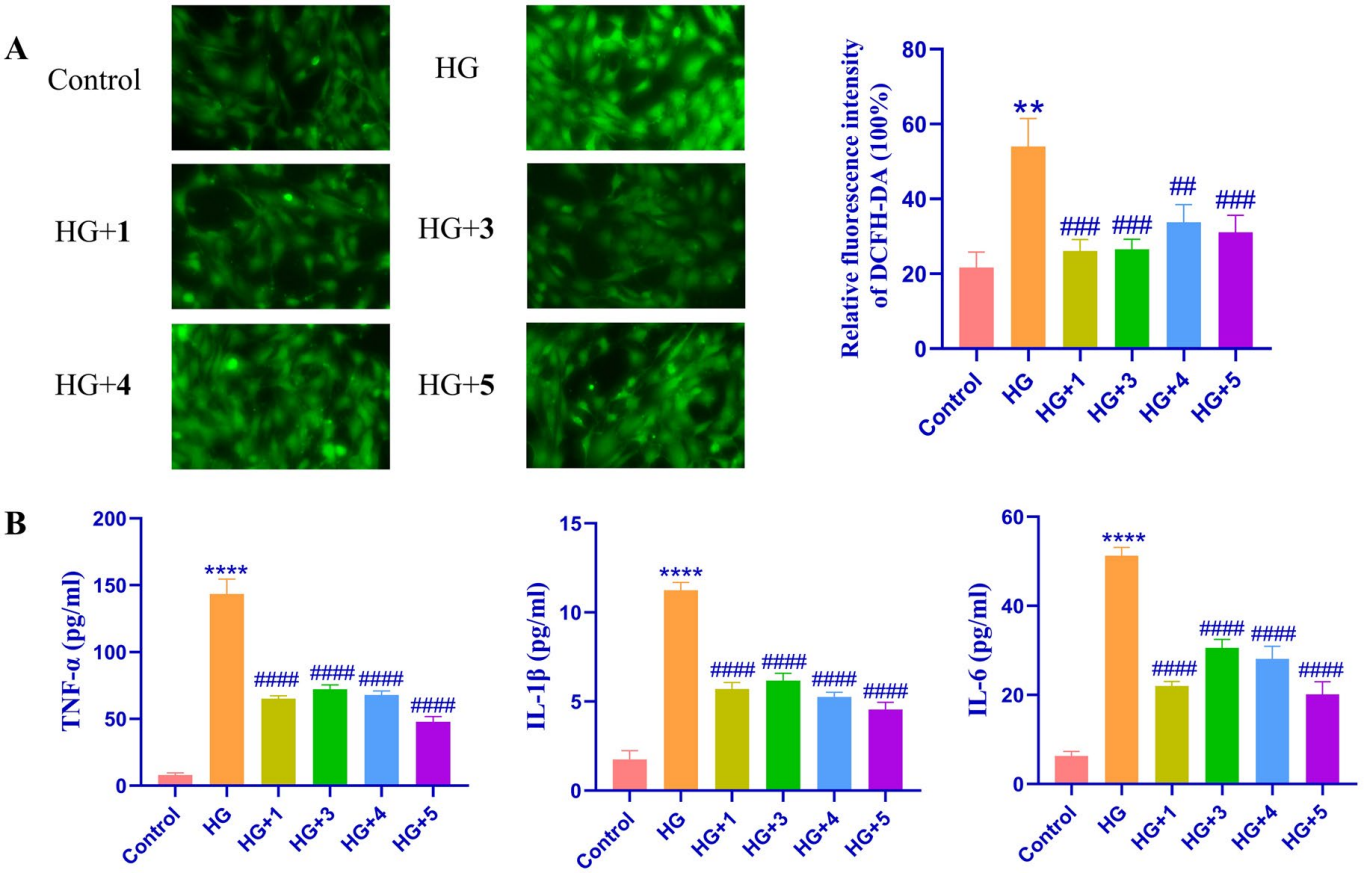
**(A)** Effect of compounds **1**, **3**, **4**, and **5** on ROS production in HBZY-1 cells under high glucose conditions. **(B)** Effects of compound **1**, **3**, **4** and **5** on ELISA results of TNF-α, IL-1β, and IL-6 in HBZY-1 cells under high glucose conditions. Data are presented as means ± SEMs (*n* = 3). **p < 0.01, vs. The control group, ****p < 0.0001, vs. The control group, ##*p* < 0.01, vs. The HG group, ###*p* < 0.001, vs. The HG group, ####*p* < 0.0001, vs. The HG group.

High glucose stimulation enhances reactive oxygen species (ROS) production, with excessive ROS accumulation driving epithelial-mesenchymal transition (EMT) development (Tian et al. 2025). Concurrently, inflammasome-mediated inflammatory responses are established contributors to diabetic complications (Tian et al. 2025). Immunofluorescence analysis revealed significantly elevated ROS levels in HBZY-1 cells after 24-hour high-glucose exposure (model group) compared with controls. Notably, treatment with the four cyanoglycosides (20 μM) markedly attenuated glucose-induced ROS surge (Figure 4A). Furthermore, quantitative assays demonstrated that the expression of TNF-α, IL-1β, and IL-6 was increased in the model group. However, TNF-α, IL-1β, and IL-6 levels increased much less in the four cyanoglycoside-treated groups than in the model group (Figure 4B). These findings demonstrated the dual protective efficacy of cyanoglycosides against high glucose-induced oxidative stress and inflammatory cytokine dysregulation in HBZY-1 cells.

#### Cyanoglycosides alleviated mitochondrial dysfunction in high-glucose induced HBZY-1 cells

**Figure 5.**
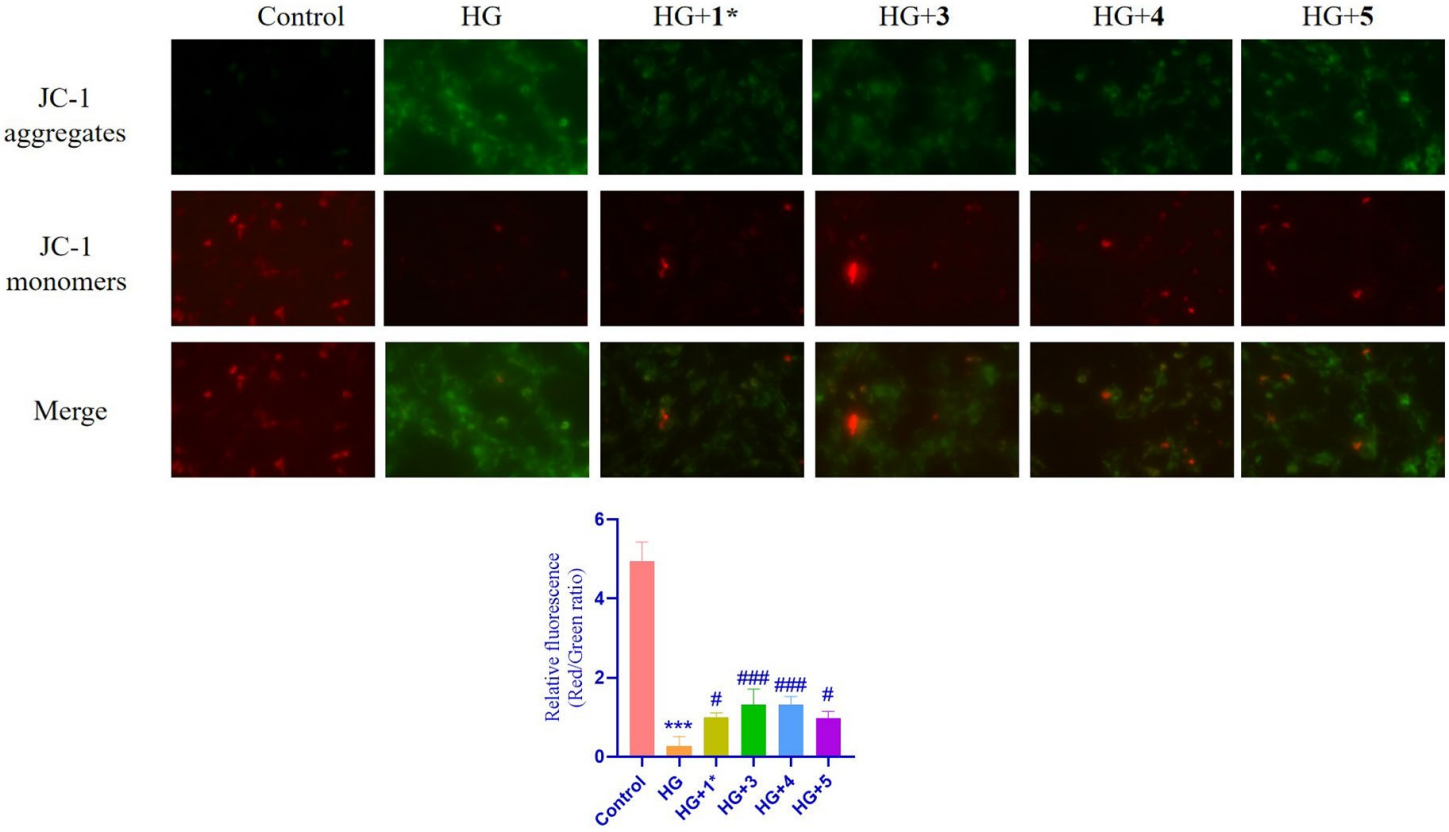
Effects of compound **1**, **3**, **4** and **5** on fluorescence microscope images of mitochondria (red) in HBZY-1 cells under high glucose conditions. Data are presented as means ± SEMs (*n* = 3). ***p < 0.001, vs. The control group, #*p* < 0.05, vs. The HG group, ###*p* < 0.001, vs. The HG group.

Under physiological conditions, reactive oxygen species (ROS), a byproduct of mitochondrial adenosine triphosphate (ATP) generation, stimulate physiological responses. Uncontrolled ROS generation damages functional proteins, causes mitochondrial dysfunction, and causes cell death (Li et al. 2013). Mitochondrial Membrane Potential (MMP) is the electrical potential difference across the inner mitochondrial membrane, and is essential for cellular energy production and various mitochondrial functions (Hao et al. 2024). MMP production was assessed to examine the changes in mitochondrial function. To assess glucose-induced mitochondrial impairment, JC-1 staining revealed significant MMP depletion in high glucose-treated HBZY-1 cells, as evidenced by the reduced red/green fluorescence ratio (indicating diminished JC-1 aggregates). Treatment with the four cyanoglycosides significantly increased the green fluorescence intensity compared with that in the high-glucose group (*p* < 0.1 or 0.001), indicating enhanced MMP (Figure 5). These findings confirm the mitochondrial protective effects of the compounds under high glucose stress.

#### Cyanoglycosides potentially ameliorated diabetic nephropathy in high-glucose-induced HBZY-1 cells through inhibition of the PFKFB3/TGF-β/Smad2/3 pathway

**Figure 6.**
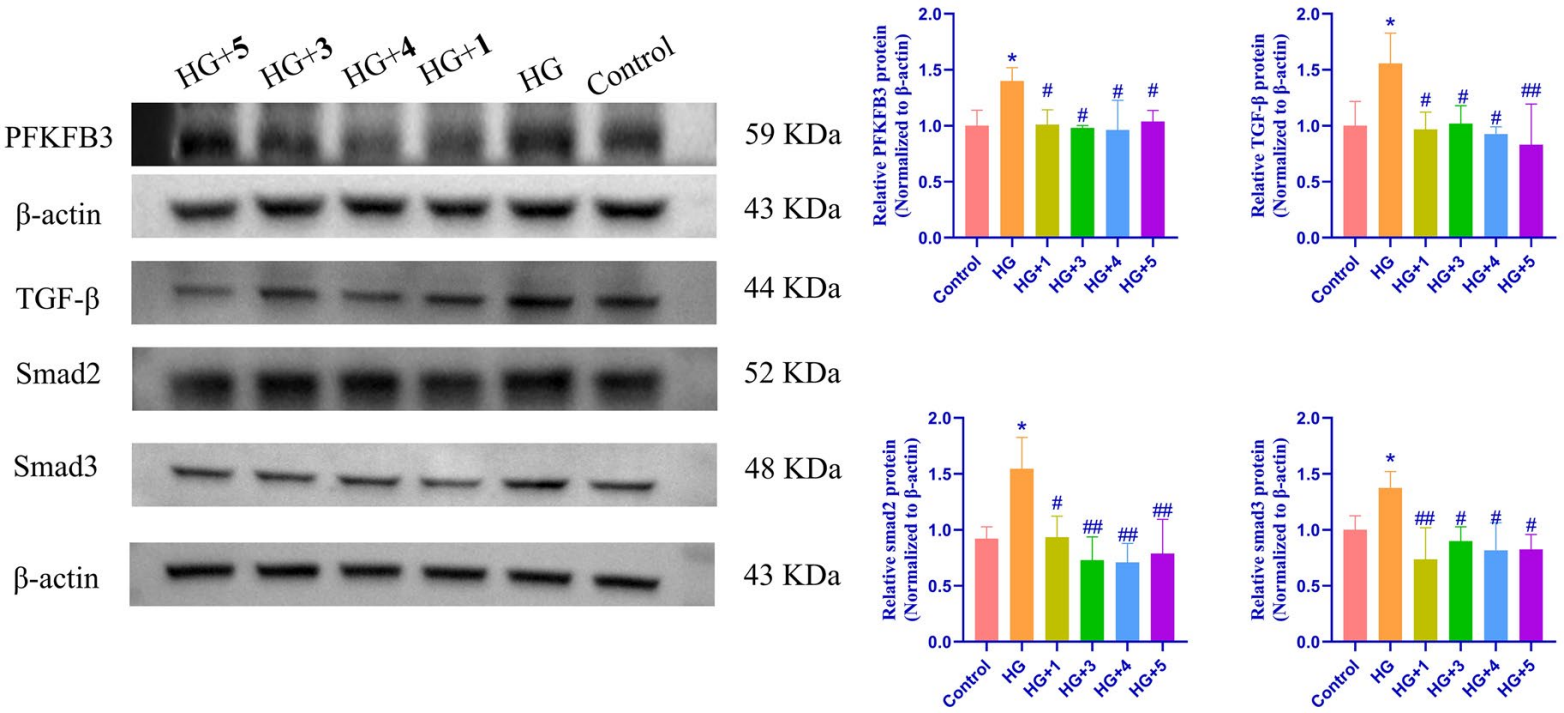
Effects of compound **1**, **3**, **4** and **5** on the expression of PFKFB3, TGF-β, Smad2 and Smad3 in HBZY-1 cells under high glucose conditions. (A) Western blot was used to detect the protein expression of PFKFB3, TGF-β, Smad2 and Smad3 in the control group, HG group and **1**, **3**, **4**, and **5** treatment group; (B) the relative protein levels of PFKFB3, TGF-β, Smad2 and Smad3. The data are expressed as average ± SEM (*n* = 3), *p < 0.05, vs. The control group, #*p* < 0.05, vs. The HG group, ##*p* < 0.01, vs. The HG group.

To further investigate the mechanism of action of the four cyanoglycosides in diabetic nephropathy, HBZY-1 cells were treated with high, followed by a 24-hour treatment with the four cyanoglycosides. Western blotting was performed to evaluate the role of PFKFB3 in glycolysis regulation *in vitro*. The expression level of PFKFB3 was significantly higher in the high-glucose treatment group than in the control group. Moreover, increased protein expression of TGF-β, Smad2, and Smad3 was also observed. Conversely, compared with the model group (the high-glucose group), treatment with the four cyanoglycosides resulted in significant decreases in the expression of the glycolysis-related protein PFKFB3 and the renal fibrosis-related proteins TGF-β, Smad2, and Smad3 (*p* < 0.1 or 0.01). Collectively, these data indicated that PFKFB3 contributes to the decreased glycolytic capacity potential mediated by TGF-β/Smad2/3 signaling in HBZY-1 cells (Figure 6).

## Conclusions

In summary, two undescribed cyanoglycosides (cyanoleiferas A and B) and three known analogs were isolated from *M. oleifera* seeds. In high glucose-induced HBZY-1 cells, the protective activities and mechanisms of action of these compounds against diabetic nephropathy were investigated. These compounds reduced the production of ROS and pro-inflammatory cytokines (TNF-α, IL-1β, and IL-6) and alleviated the decrease in mitochondrial membrane potential. Western blot analysis showed that cyanoglycosides effectively decreased the expression of glycolysis-related enzyme PFKFB3. Cyanoglycosides significantly reduce the expression of renal fibrosis-related proteins (TGF-β/Smad2/Smad3). These results indicate that cyanoglycosides may alleviate diabetic nephropathy by driving metabolic reprogramming and the potential PFKFB3/TGF-β/Smads pathway. Through their unique α-hydroxynitrile structure, nitrile glycosides simultaneously regulate metabolic abnormalities (PFKFB3-mediated glycolysis) and fibrosis processes (the TGF-β/Smad pathway). Derived from medicinal and edible plants, these compounds have good clinical acceptance. Their clear structures are advantageous for quality control and structural modification, thereby demonstrating their therapeutic potential in DN. However, due to the limited availability of some isolates, only compounds **1**, **3**, **4**, and **5** were obtained in sufficient quantities for in-depth mechanistic *in vitro* studies. Nevertheless, this limitation does not compromise the significance of our evaluation of the anti-diabetic nephropathy activity of this class of compounds. Additionally, the protective effects of cyanoglycosides on diabetic nephropathy require further *in vivo* investigation following total synthesis.

However, this study has some limitations; for example, the *in vitro* cell model (HBZY-1) cannot fully simulate the renal microenvironment *in vivo*, and there is a gap between the single injury model induced by high glucose and the multifactorial pathology of clinical DN. The absorption/distribution/metabolism parameters of compounds in organisms are not yet clear, and the metabolic transformation of cyanogenic glycosides in intestinal microbiota may affect their active forms. Although the regulatory effect of PFKFB3 was confirmed by protein expression, real-time monitoring of glycolytic flux is lacking. This needs to be verified by further validation using animal models and in-depth research on this mechanism.

## Supplementary Material

SI_revised_20251121 (2).docx

## Data Availability

The data presented in this study are available in Supplementary Materials.
