# Supplementary material for: Cyanoglycosides isolated from Moringa oleifera seeds inhibited PFKFB3/TGF-β1/smads pathway to alleviate diabetic nephropathy through driving metabolic reprogramming
Source: Pharm Biol. 2025 Dec 29;64(1):130–42. doi: 10.1080/13880209.2025.2607563 (PMC12777776; doi:10.1080/13880209.2025.2607563)
Supplement: SI_revised_20251121 (2).docx [file IPHB_A_2607563_SM2966.docx]

**Cyanoglycosides isolated from *Moringa oleifera* seeds inhibited PFKFB3/TGF-β1/Smads pathway to alleviate diabetic nephropathy through driving metabolic reprogramming**

Chengyu Ge^1,2,a^, Zhihua Shi^3,a^, Jia He^1,2^, Xu Feng^1,2^, Kaiqi Shang^1,2^, Xiaolin Liao^3^, Yufeng Liu^4^, Yueping Jiang^1,2,3,5^^*^, Shao Liu^1,2*^

^1^Department of Pharmacy, Xiangya Hospital, Central South University, Changsha, 410008, Hunan, China.

^2^National Clinical Research Center for Geriatric Disorders, Xiangya Hospital, Central South University, Changsha, 410008, Hunan, China.

^3^College of Pharmacy, Jining Medical University, Rizhao, 276826, Shandong, China.

^4^Department of Clinical Pharmacy, Hunan University of Medicine General Hospital, Huaihua, 418000, Hunan, China.

^5^Hunan Provincial Key Laboratory of the Research and Development of Novel Pharmaceutical Preparations, “The 14th Five-Year Plan” Application Characteristic Discipline of Hunan Province (Pharmaceutical Science), College of Pharmacy, Changsha Medical University, Changsha, 410219, Hunan, China.

^a^ These authors contributed equally to this work.

*Corresponding authors: Yueping Jiang, E-mial:[jiangyueping@csu.edu.cn](mailto:jiangyueping@csu.edu.cn); No. 87 Xiangya Road, Changsha city, 410008, Hunan province, China; Tel: +86 15111052919.

Shao Liu, E-mial: liushao999@csu.edu.cn; No. 87 Xiangya Road, Changsha city, 410008, Hunan province, China; Tel: +86 13723873434.

**Supporting Information**

**List of Contents**

| No. | Contents | Page |
| --- | --- | --- |
| 1 | **Figure S1**. The UV spectrum of compound **1** in MeOH. | S3 |
| 2 | **Figure S2**. The IR spectrum of compound **1**. | S3 |
| 3 | **Figure S3**. The (+)-HRESIMS report of compound **1**, page 1. | S4 |
| 4 | **Figure S4**. The (+)-HRESIMS report of compound **1**, page 2. | S5 |
| 5 | **Figure S5**. The ^1^H NMR spectrum of compound **1** in MeOH-*d*_4_ (600 MHz). | S6 |
| 6 | **Figure S6**. The ^13^C NMR spectrum of compound **1** in MeOH-*d*_4_ (150 MHz). | S6 |
| 7 | **Figure S7**. The DEPT spectrum of compound **1** in MeOH-*d*_4_ (150 MHz). | S7 |
| 8 | **Figure S8**. The ^1^H-^1^H COSY spectrum of compound **1** in MeOH-*d*_4_ (600 MHz). | S7 |
| 9 | **Figure S9**. The HSQC spectrum of compound **1** in MeOH-*d*_4_ (600 MHz for ^1^H). | S8 |
| 10 | **Figure S10**. The HMBC spectrum of compound **1** in MeOH-*d*_4_ (600 MHz for ^1^H). | S8 |
| 11 | **Figure S11**. The UV spectrum of compound **2** in MeOH. | S9 |
| 12 | **Figure S12**. The IR spectrum of compound **2**. | S9 |
| 13 | **Figure S13**. The (+)-HRESIMS report of compound **2**, page 1. | S10 |
| 14 | **Figure S14**. The (+)-HRESIMS report of compound **2**, page 2. | S11 |
| 15 | **Figure S15**. The ^1^H NMR spectrum of compound **2** in MeOH-*d*_4_ (600 MHz). | S12 |
| 16 | **Figure S16**. The ^13^C NMR spectrum of compound **2** in MeOH-*d*_4_ (150 MHz). | S12 |
| 17 | **Figure S17**. The DEPT spectrum of compound **2** in MeOH-*d*_4_ (150 MHz). | S13 |
| 18 | **Figure S18**. The ^1^H-^1^H COSY spectrum of compound **2** in MeOH-*d*_4_ (600 MHz). | S13 |
| 19 | **Figure S19**. The HSQC spectrum of compound **2** in MeOH-*d*_4_ (600 MHz for ^1^H). | S14 |
| 20 | **Figure S20**. The HMBC spectrum of compound **2** in MeOH-*d*_4_ (600 MHz for ^1^H). | S14 |
| 21 | **Figure S21**. The original western bolt graphs of β-actin and PFKFB3 | S15 |
| 22 | **Figure S22**. The original western blot graphs of β-actin, TGF-β, smad3, and smad2. | S15 |
| 23 | **Table S1**. The detailed information of primary and secondary antibodies used in this study. | S16 |


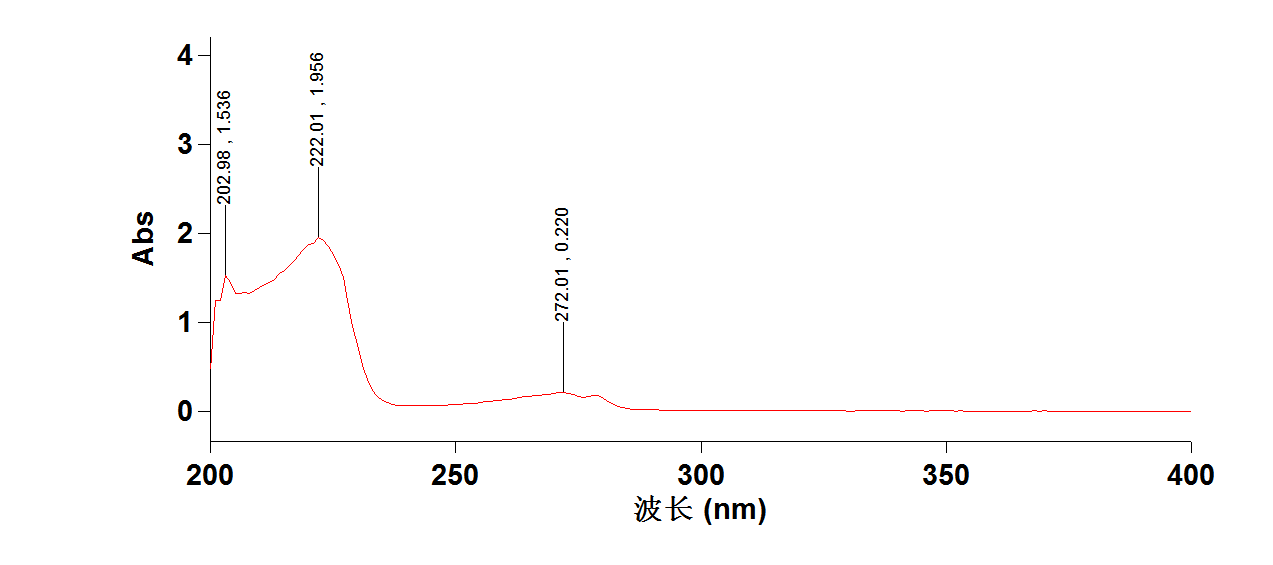


**Figure S1**. The UV spectrum of compound **1** in MeOH.


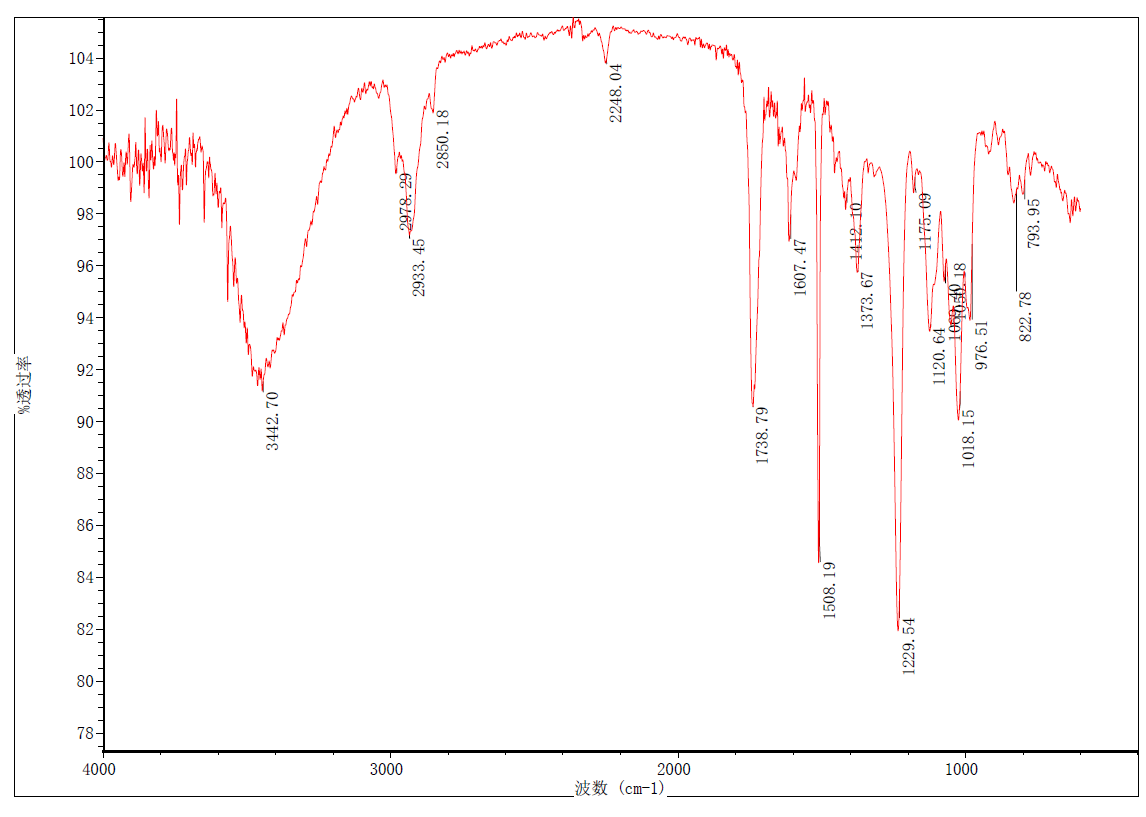


**Figure S2**. The IR spectrum of compound **1**.


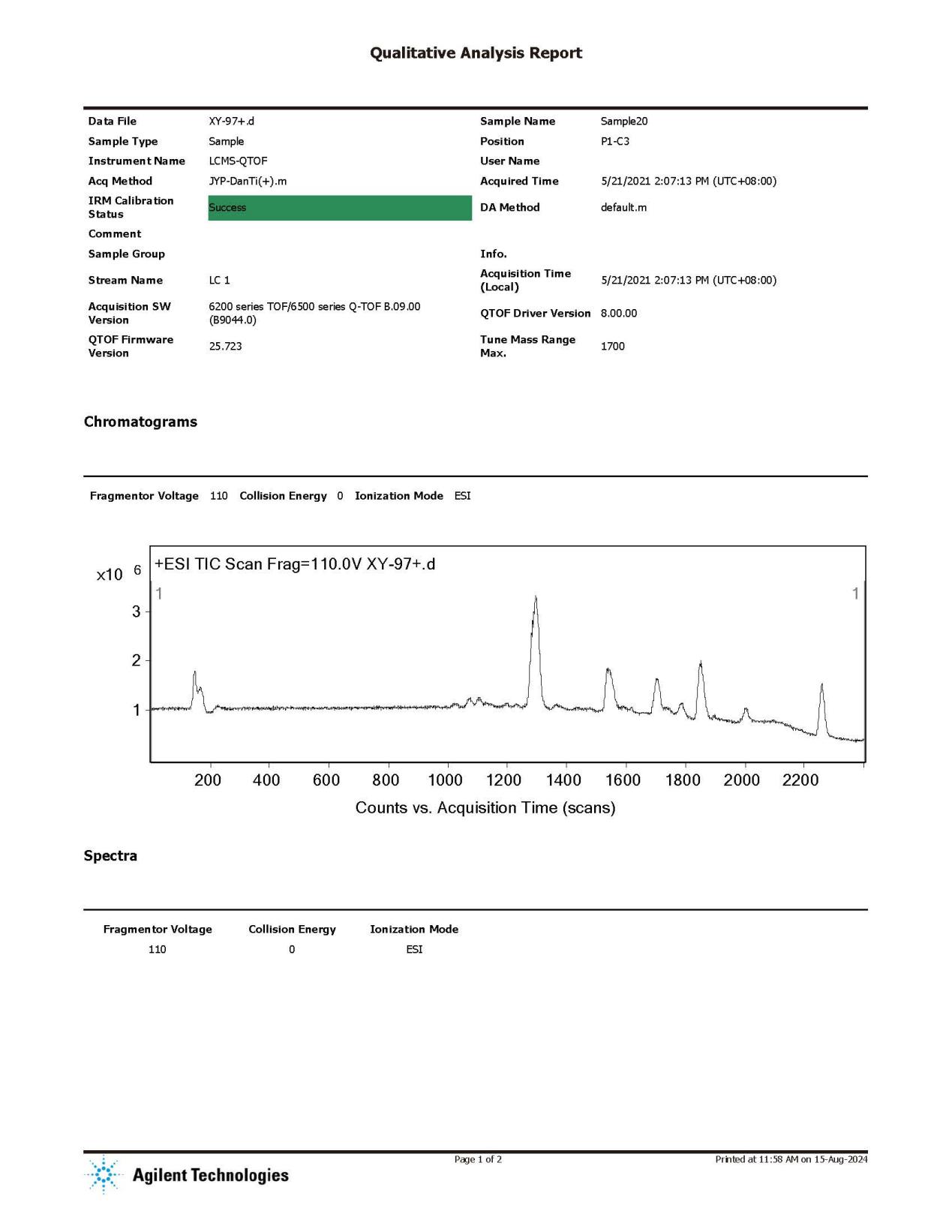


**Figure S3**. The (+)-HRESIMS report of compound **1**, page 1.


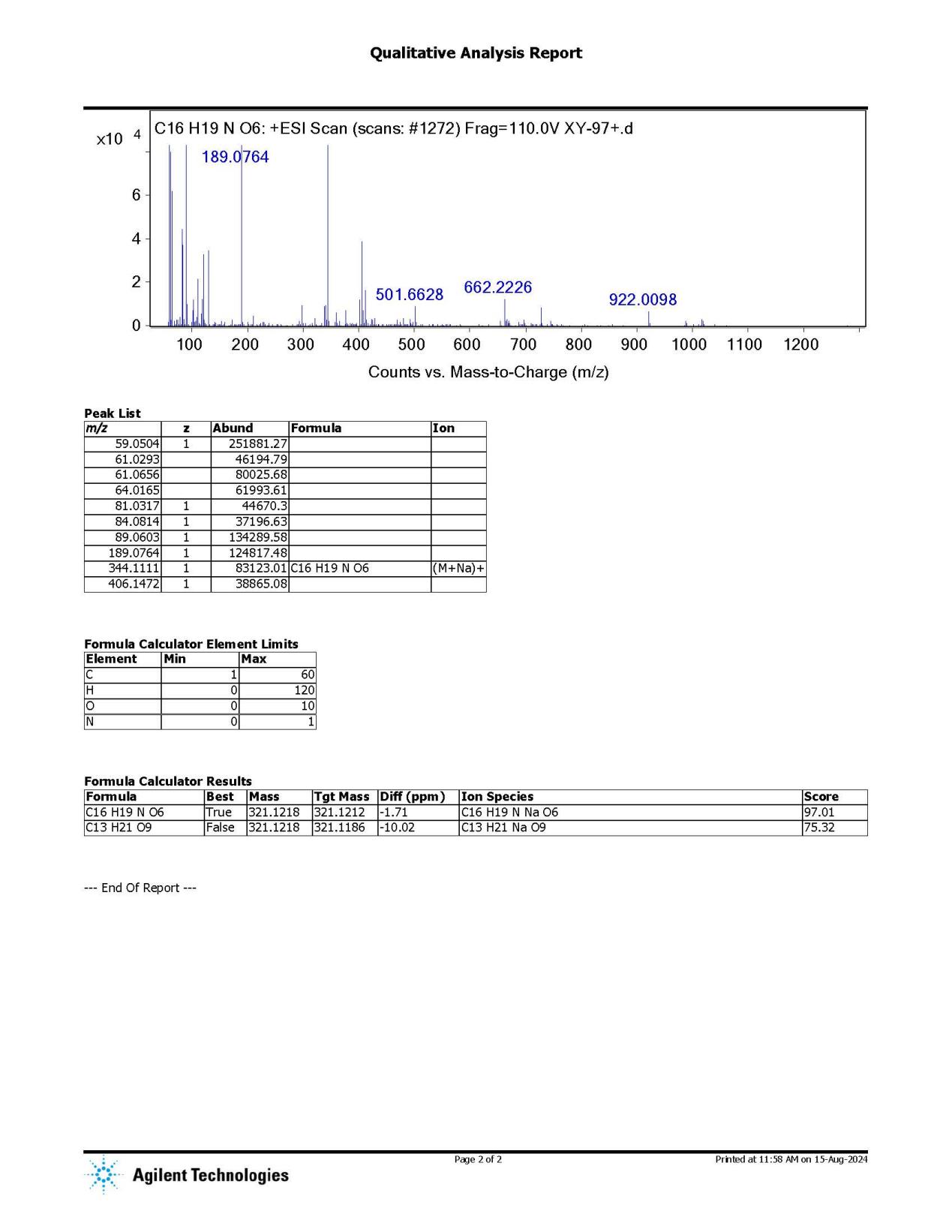


**Figure S4**. The (+)-HRESIMS report of compound **1**, page 2.


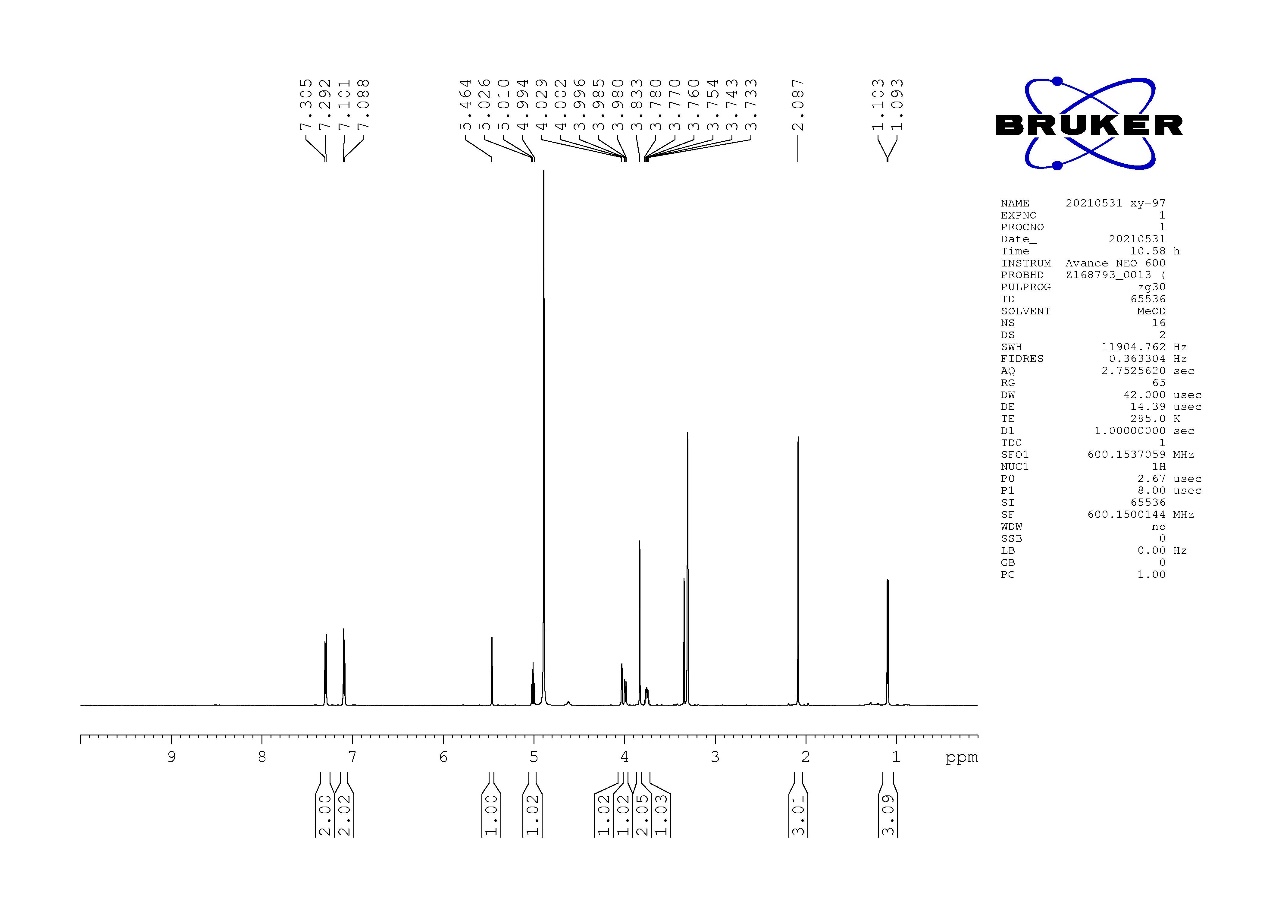


**Figure S5**. The ^1^H NMR spectrum of compound **1** in MeOH-*d*_4_ (600 MHz).


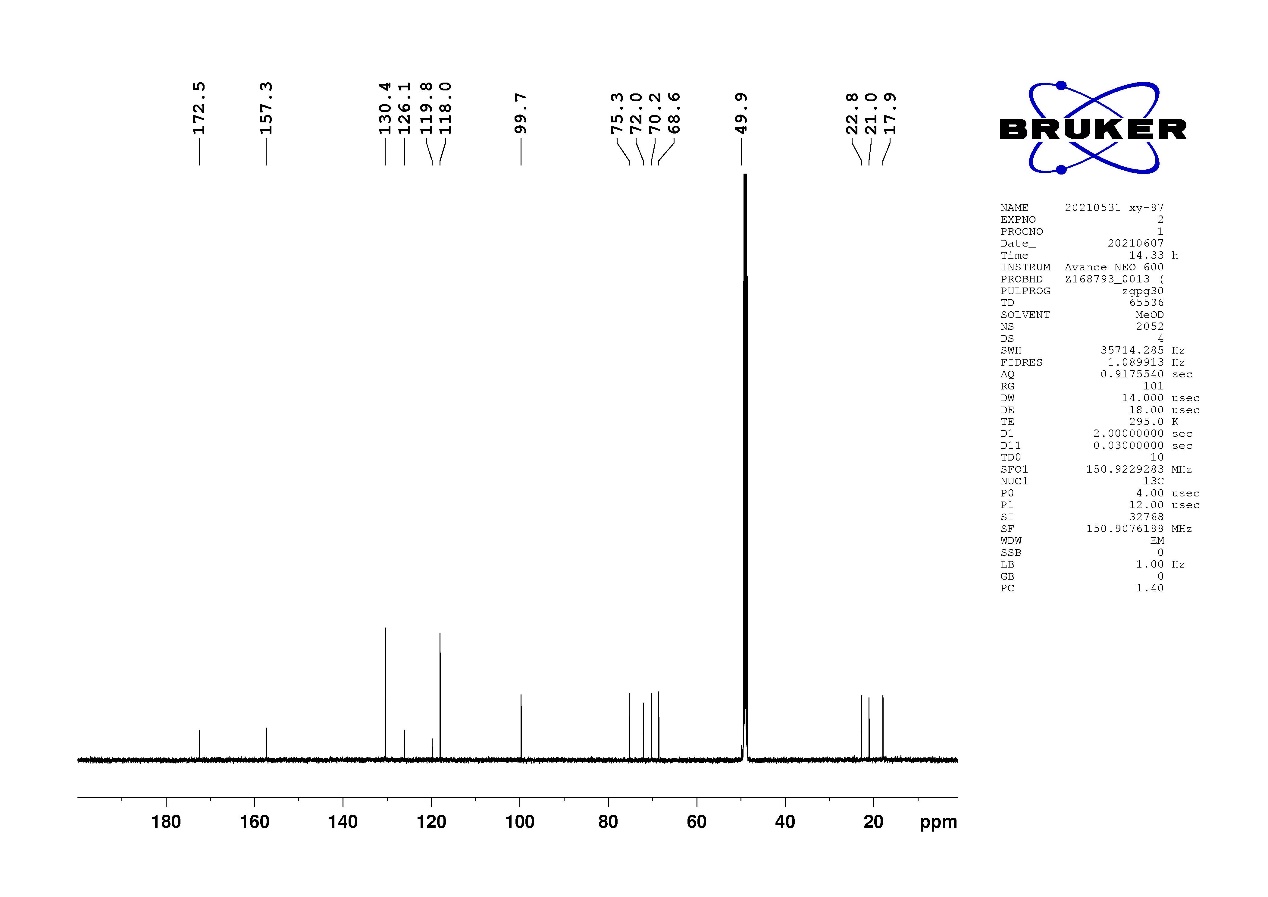


**Figure S6**. The ^13^C NMR spectrum of compound **1** in MeOH-*d*_4_ (150 MHz).


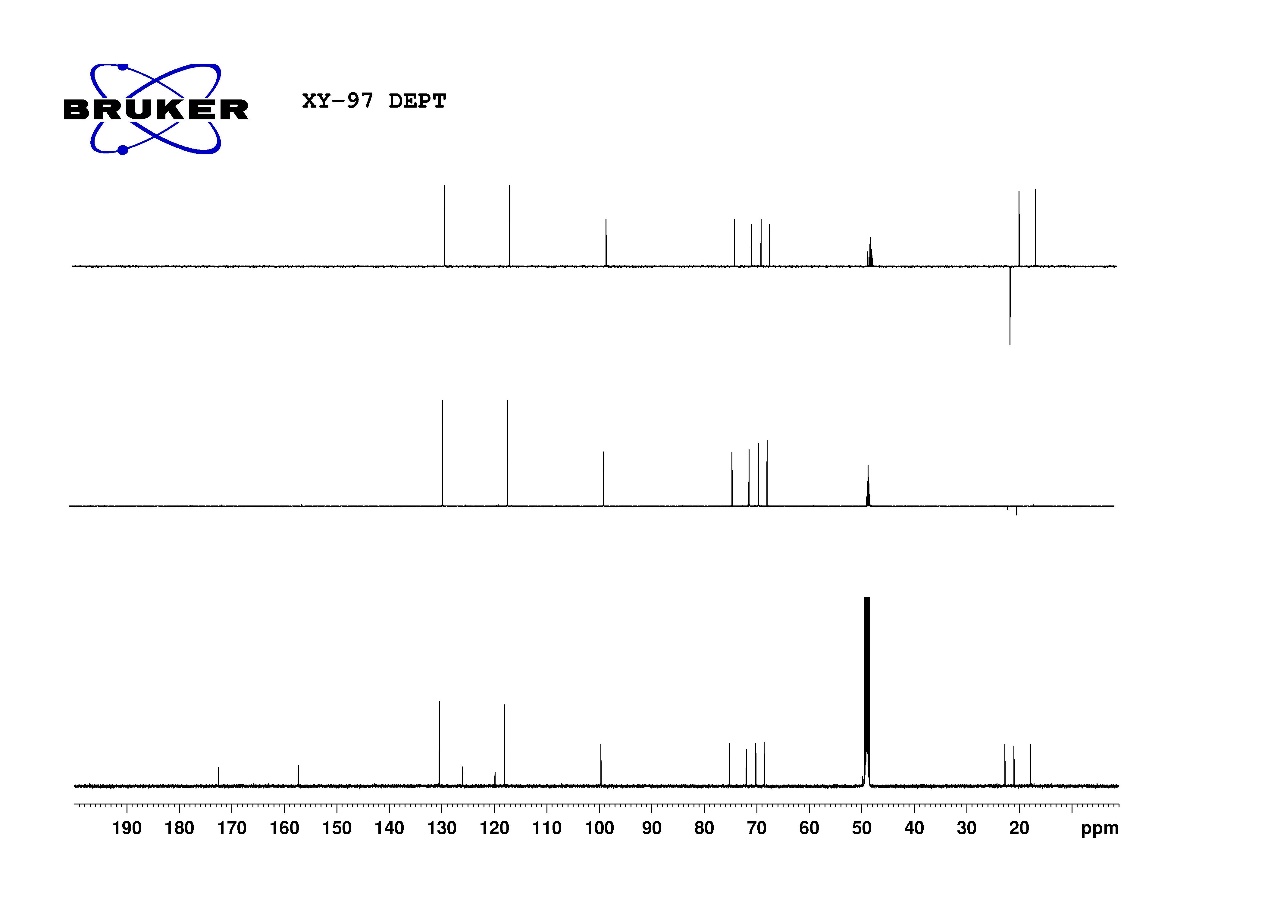


**Figure S7**. The DEPT spectrum of compound **1** in MeOH-*d*_4_ (150 MHz).


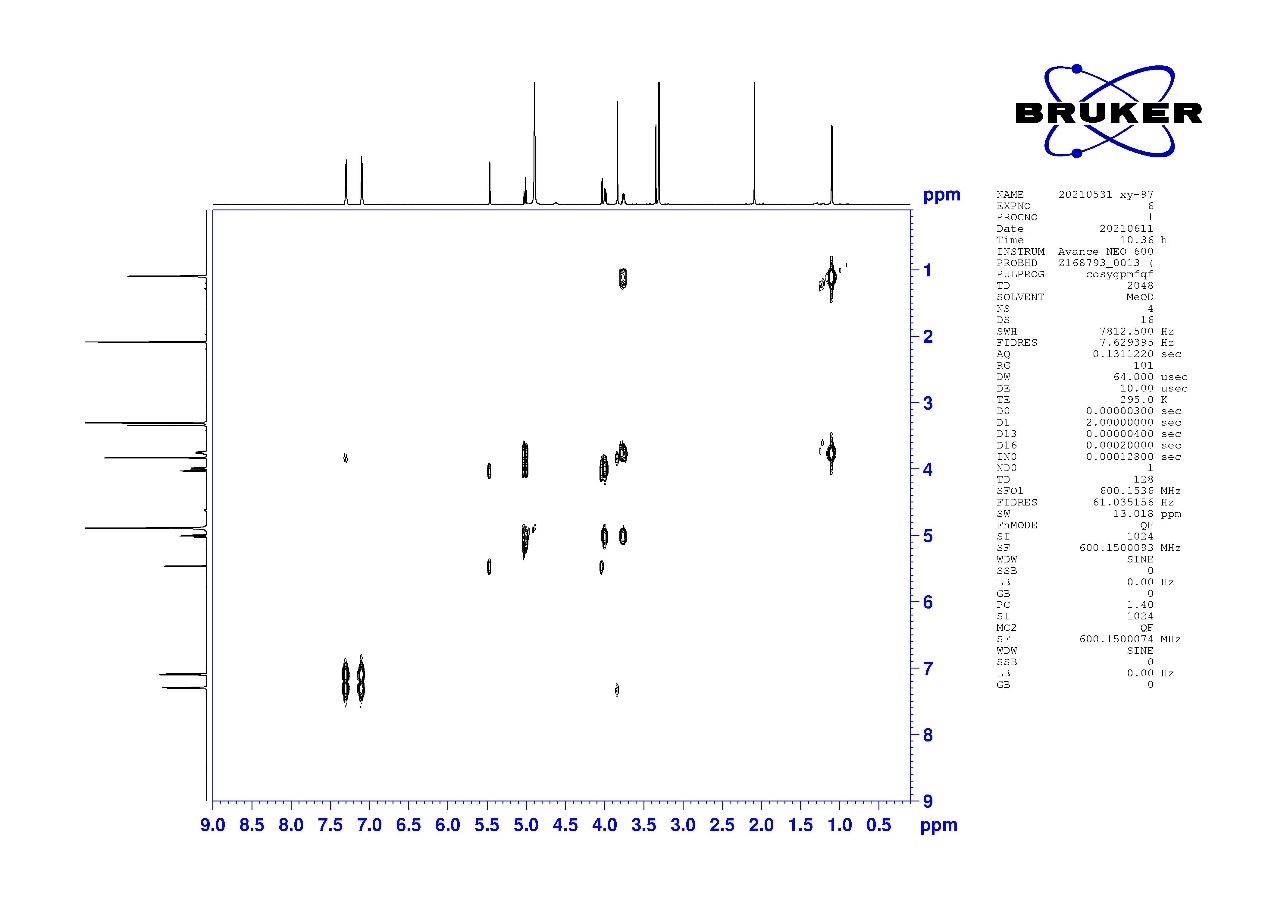


**Figure S8**. The ^1^H-^1^H COSY spectrum of compound **1** in MeOH-*d*_4_ (600 MHz).


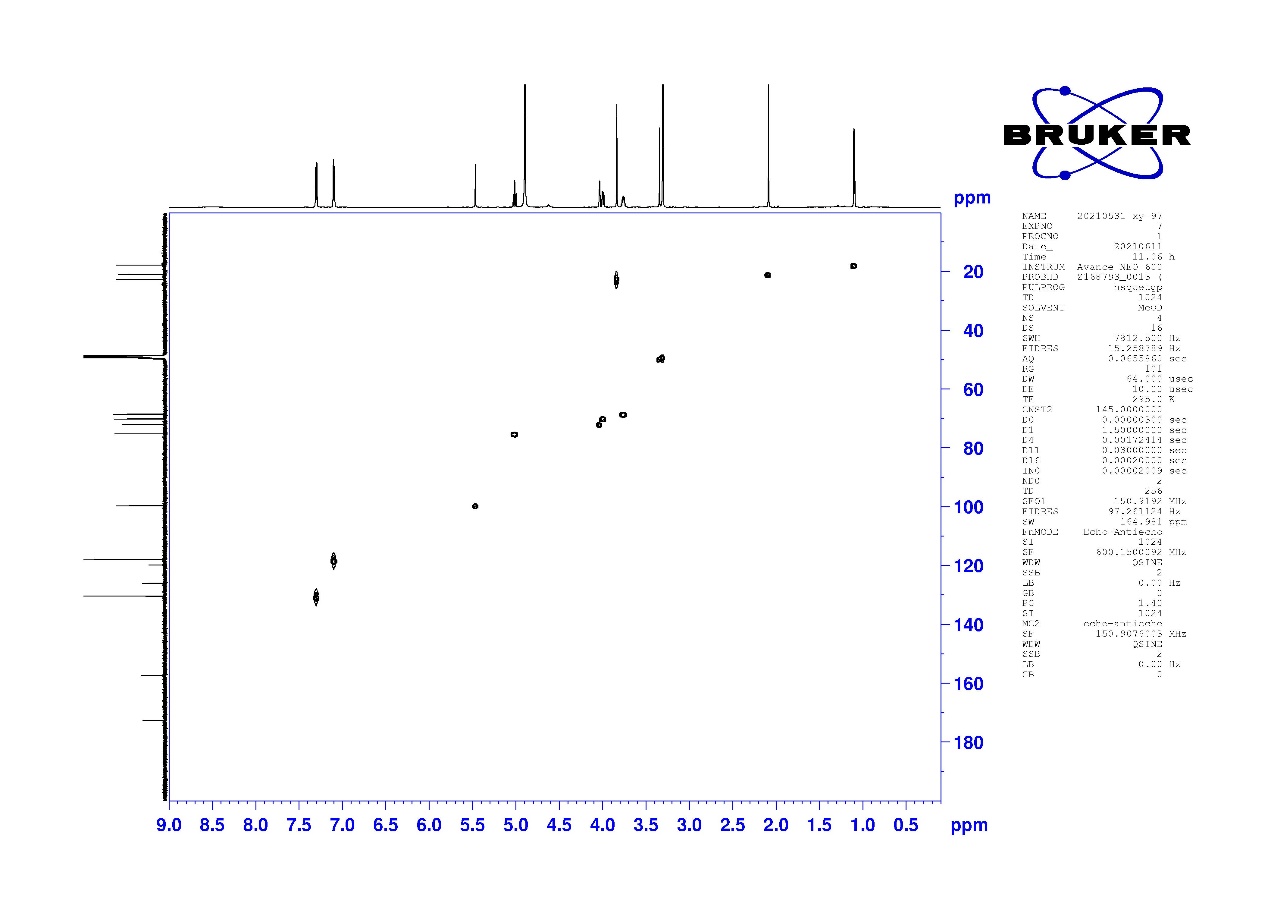


**Figure S9**. The HSQC spectrum of compound **1** in MeOH-*d*_4_ (600 MHz for ^1^H).


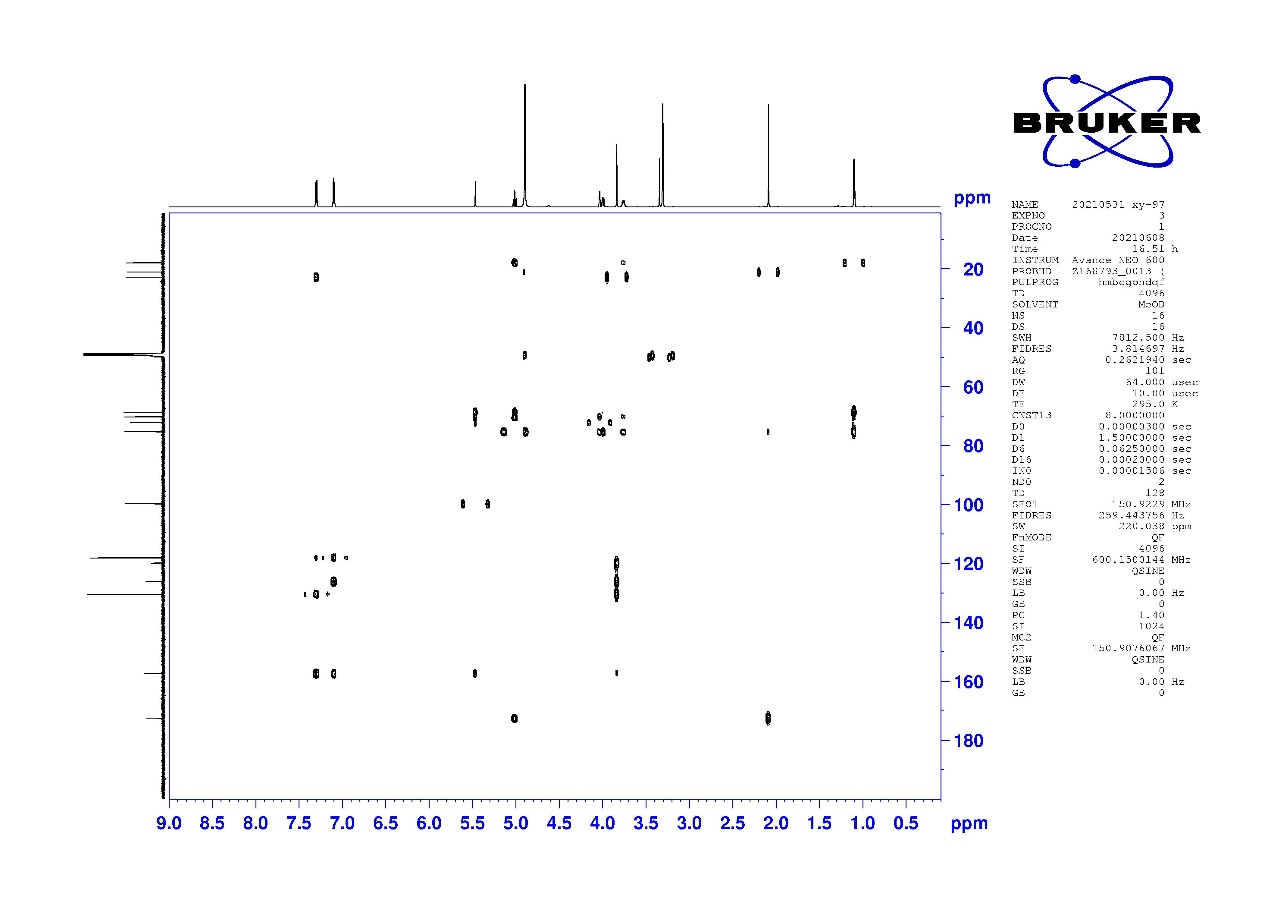


**Figure S10**. The HMBC spectrum of compound **1** in MeOH-*d*_4_ (600 MHz for ^1^H).


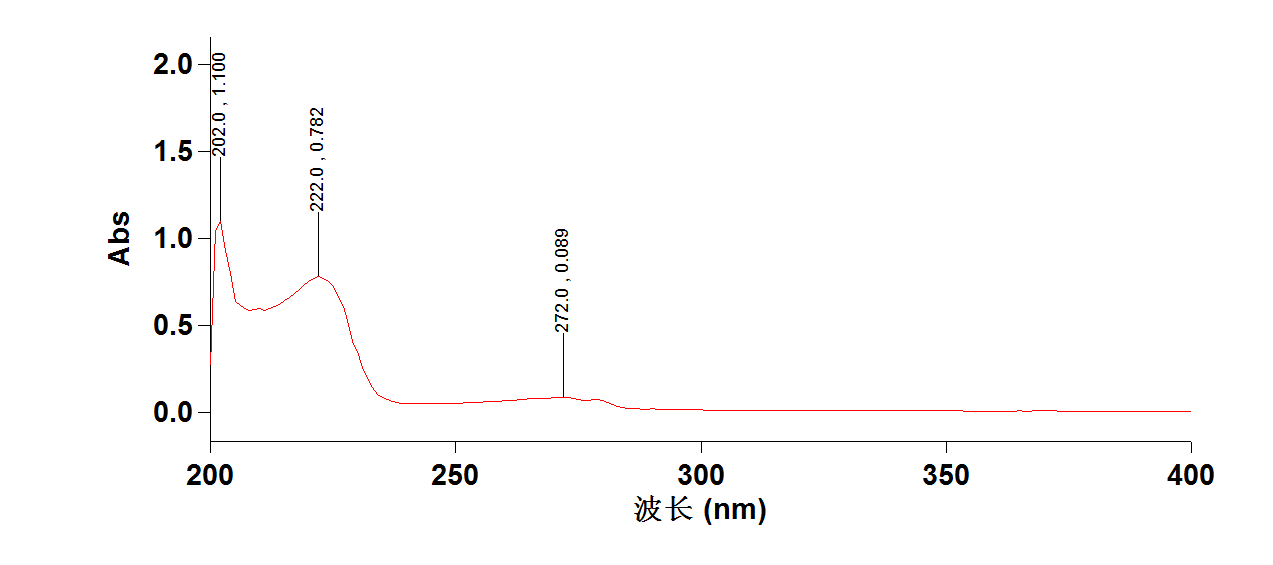


**Figure S11**. The UV spectrum of compound **2** in MeOH.


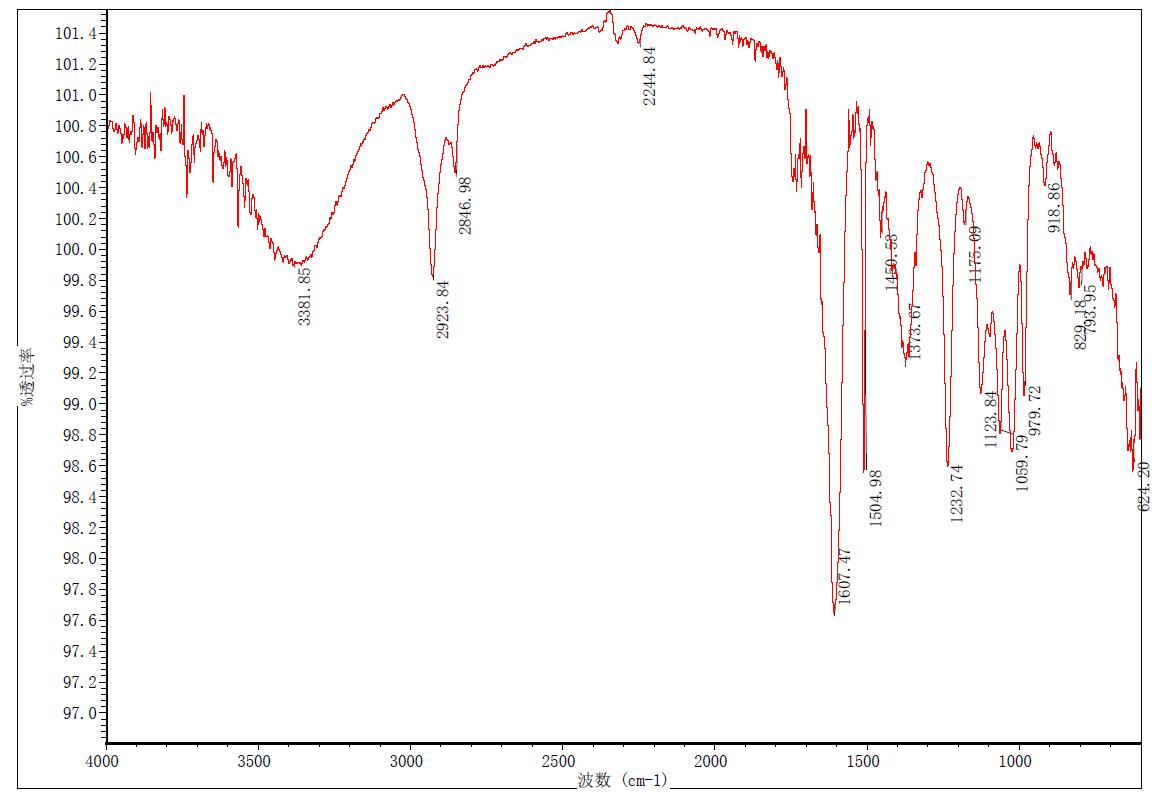


**Figure S12**. The IR spectrum of compound **2**.


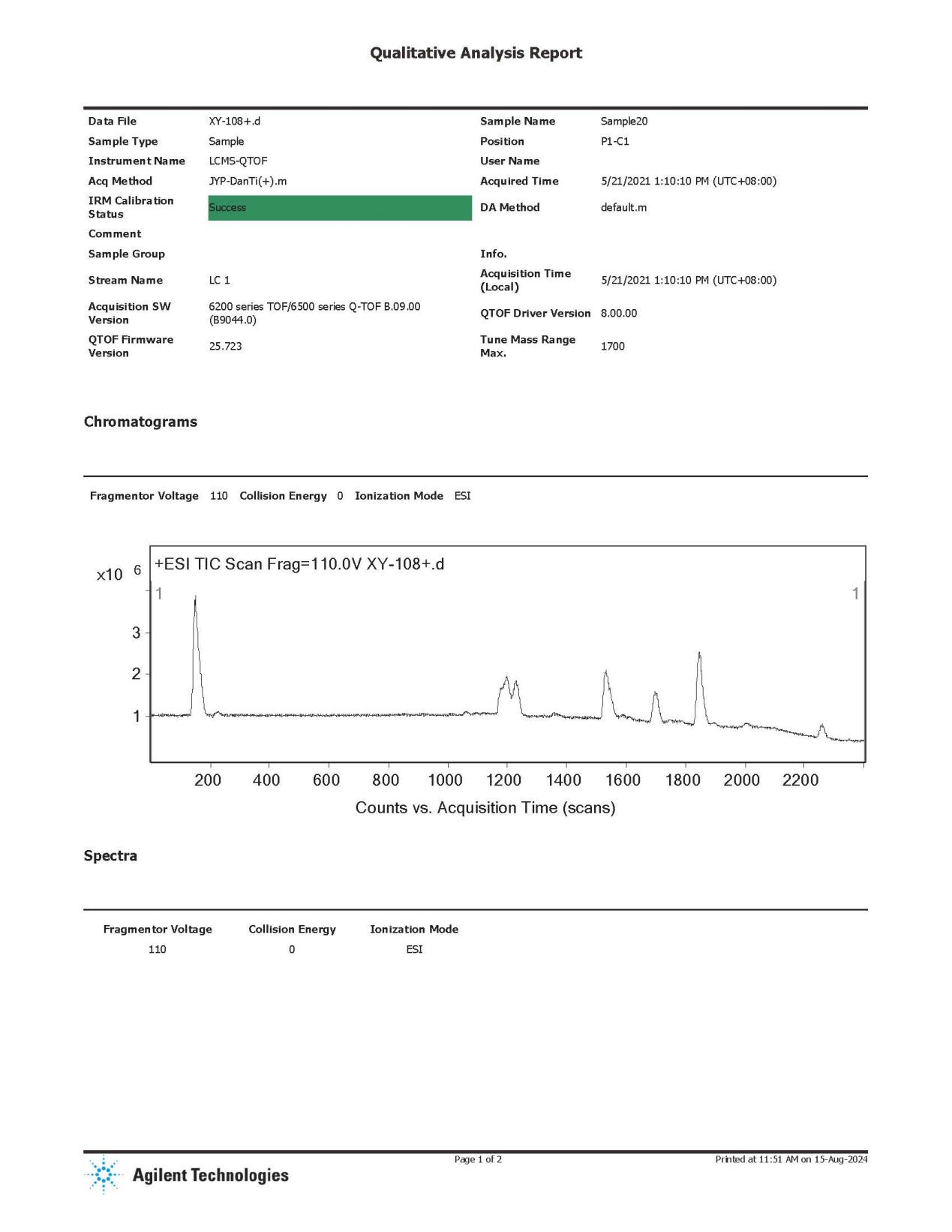


**Figure S13**. The (+)-HRESIMS report of compound **2**, page 1.


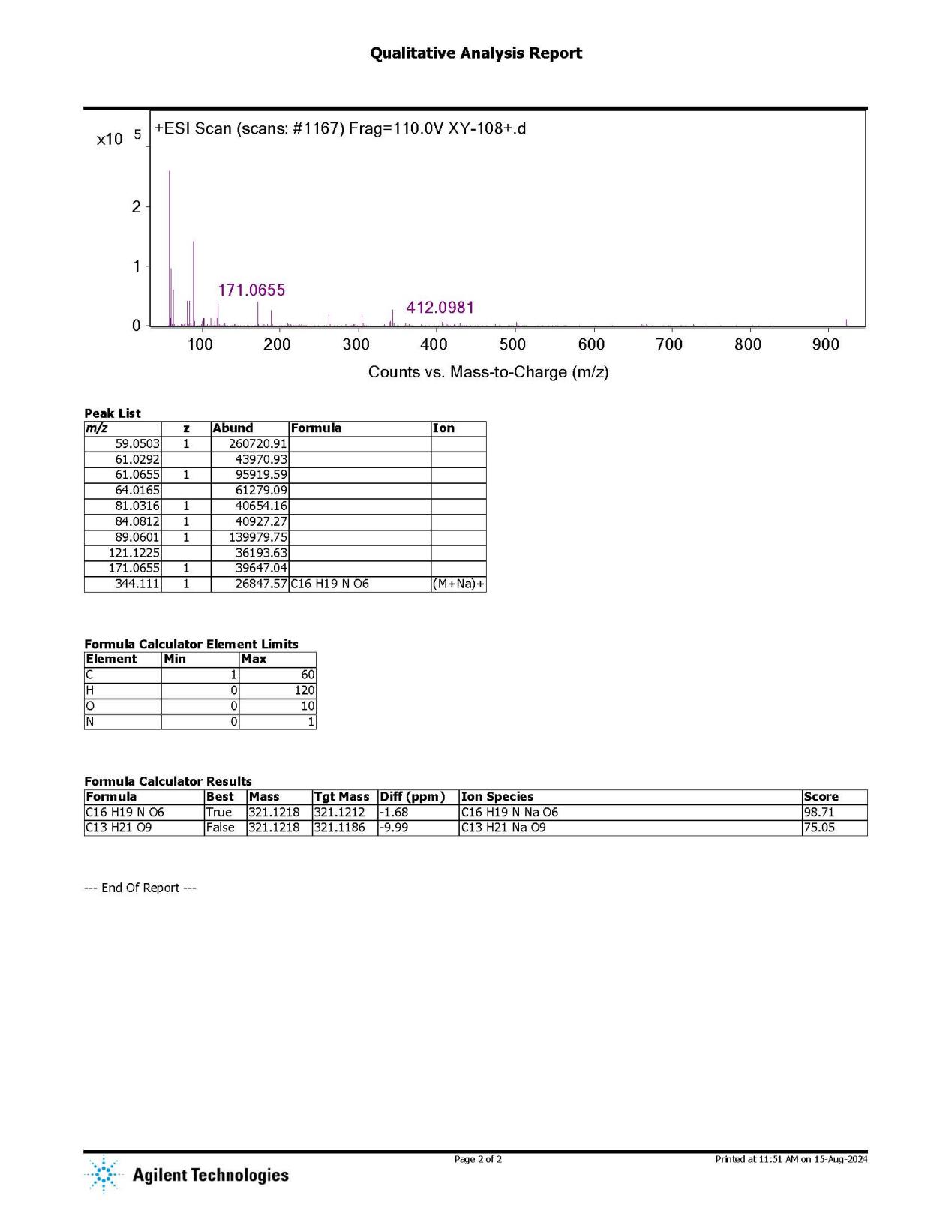


**Figure S14**. The (+)-HRESIMS report of compound **2**, page 2.


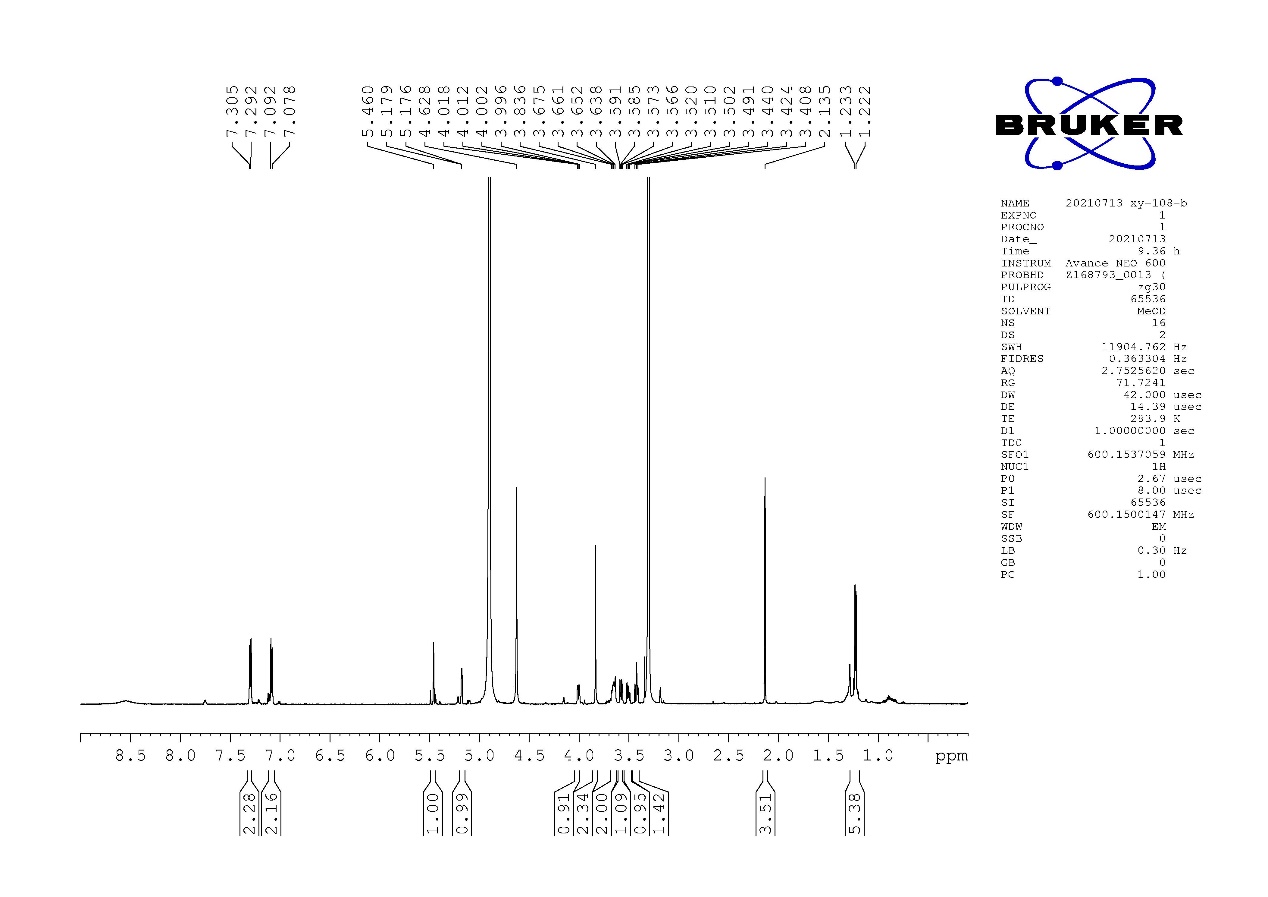


**Figure S15**. The ^1^H NMR spectrum of compound **2** in MeOH-*d*_4_ (600 MHz).


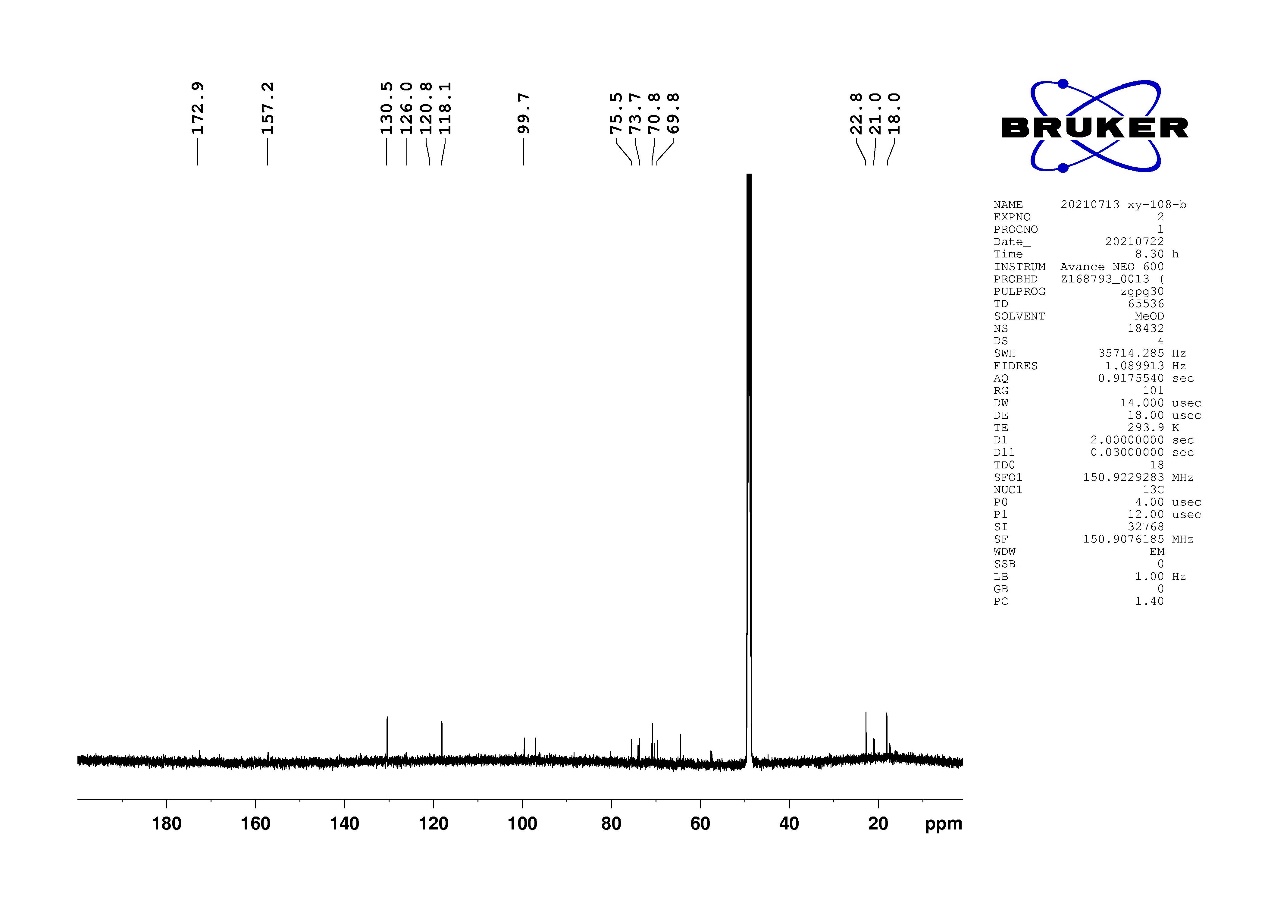


**Figure S16**. The ^13^C NMR spectrum of compound **2** in MeOH-*d*_4_ (150 MHz).


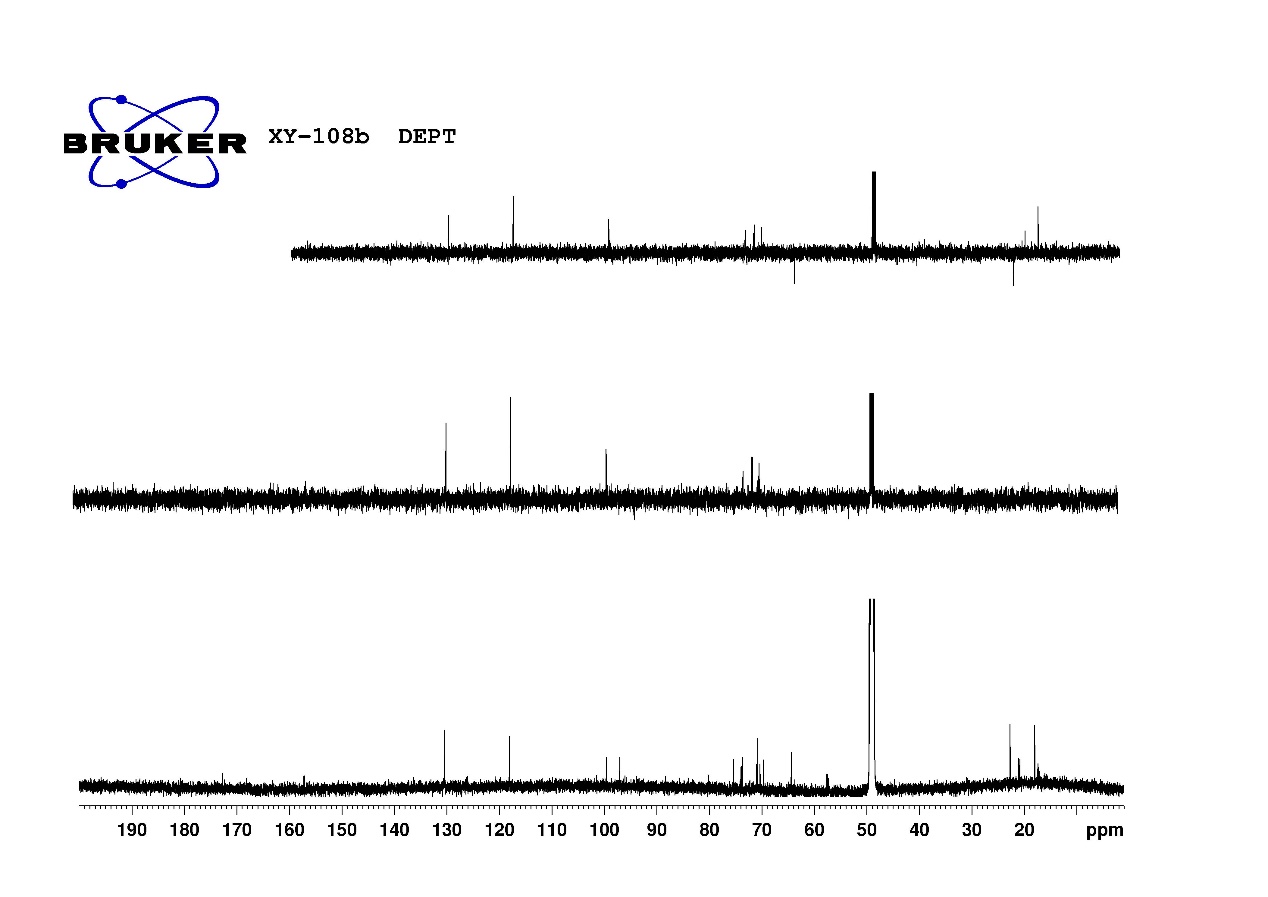


**Figure S17**. The DEPT spectrum of compound **2** in MeOH-*d*_4_ (150 MHz).


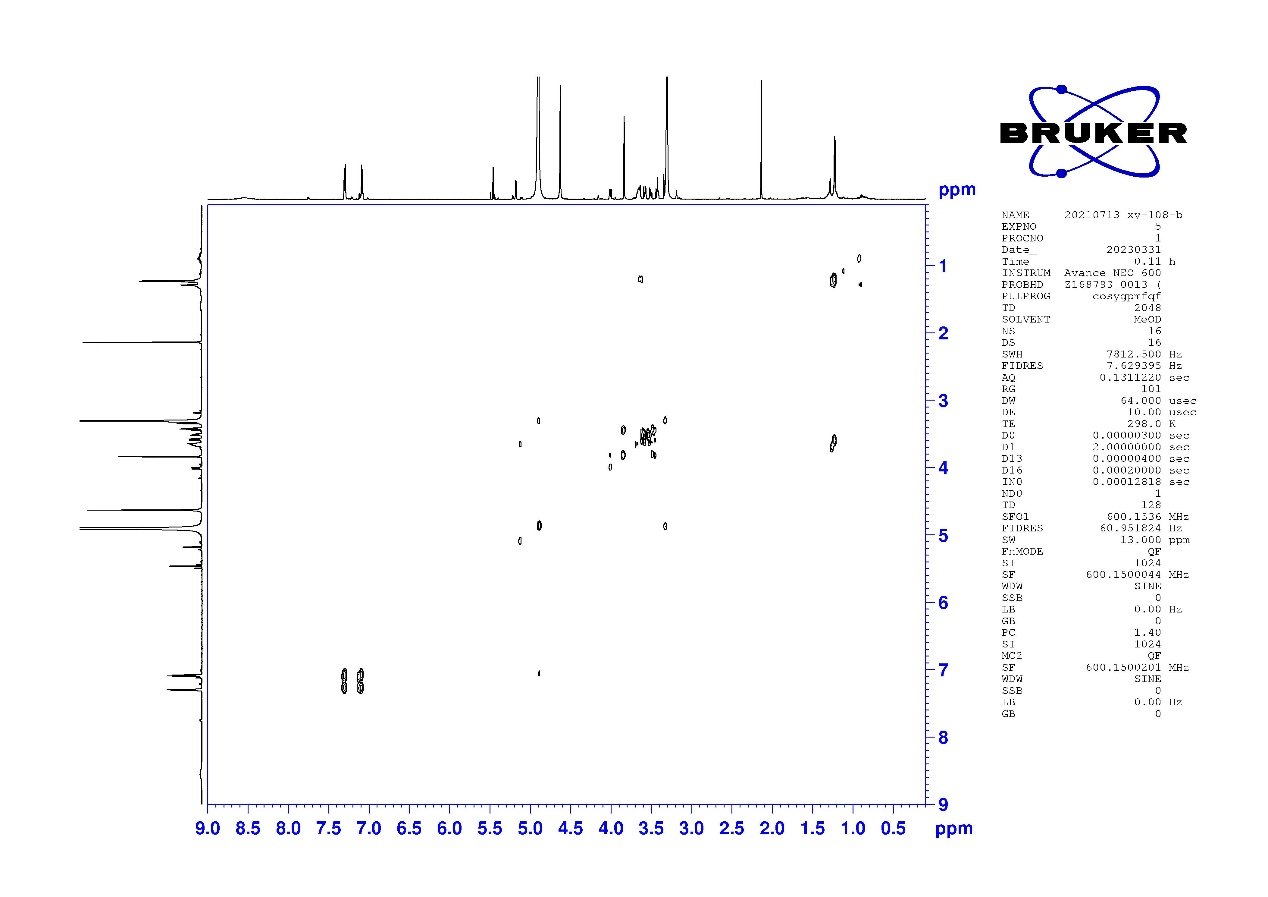


**Figure S18**. The ^1^H-^1^H COSY spectrum of compound **2** in MeOH-*d*_4_ (600 MHz).


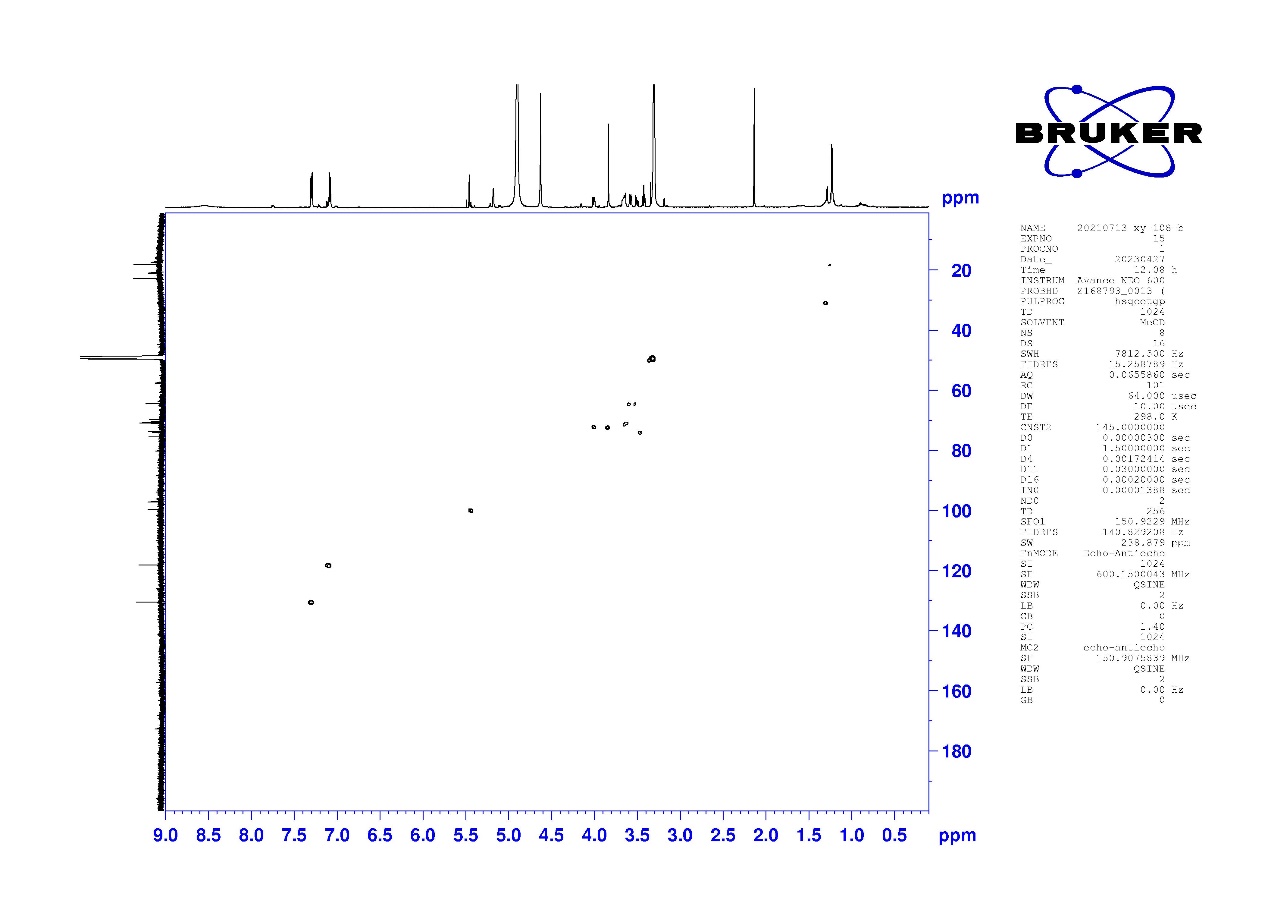


**Figure S19**. The HSQC spectrum of compound **2** in MeOH-*d*_4_ (600 MHz for ^1^H).


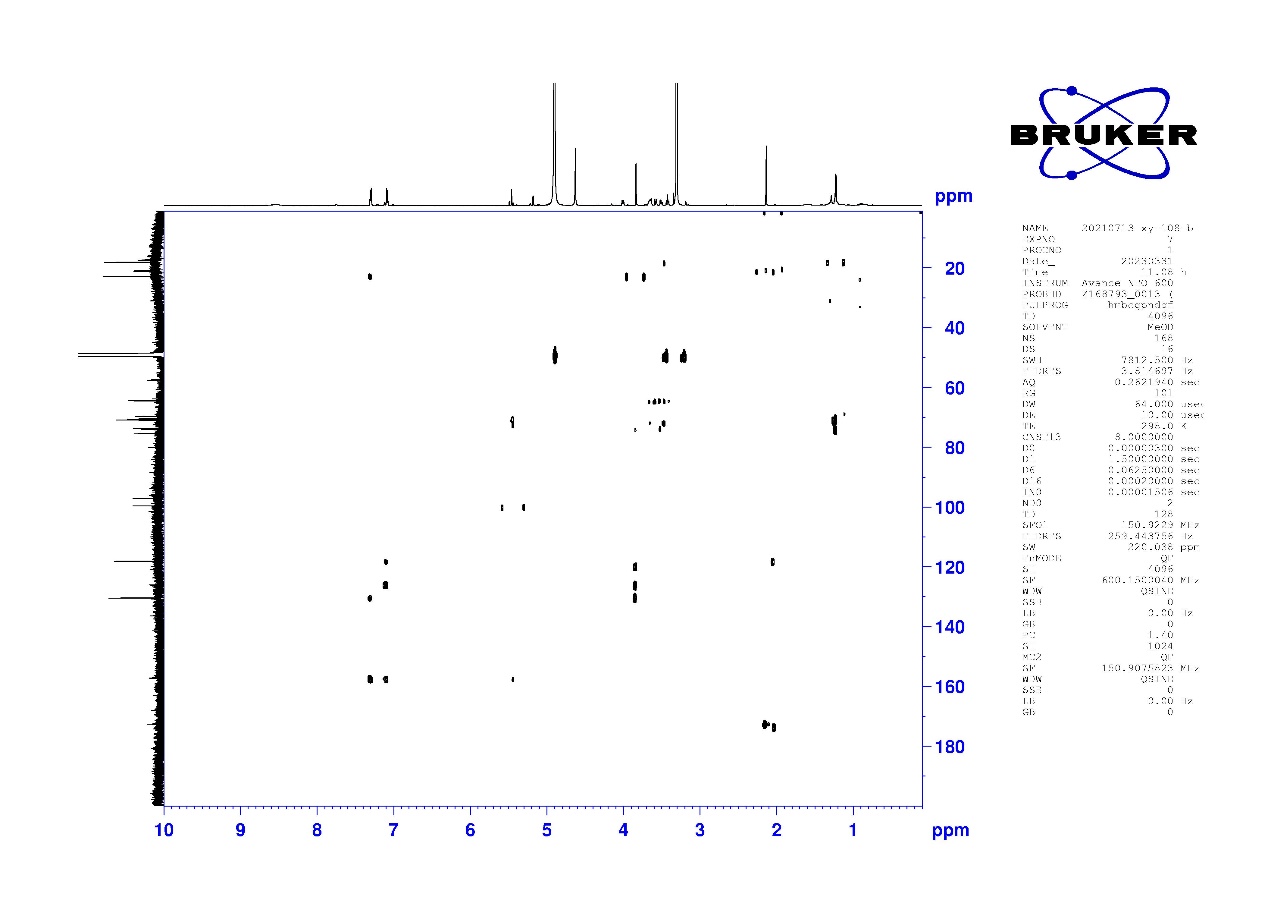


**Figure S20**. The HMBC spectrum of compound **2** in MeOH-*d*_4_ (600 MHz for ^1^H).


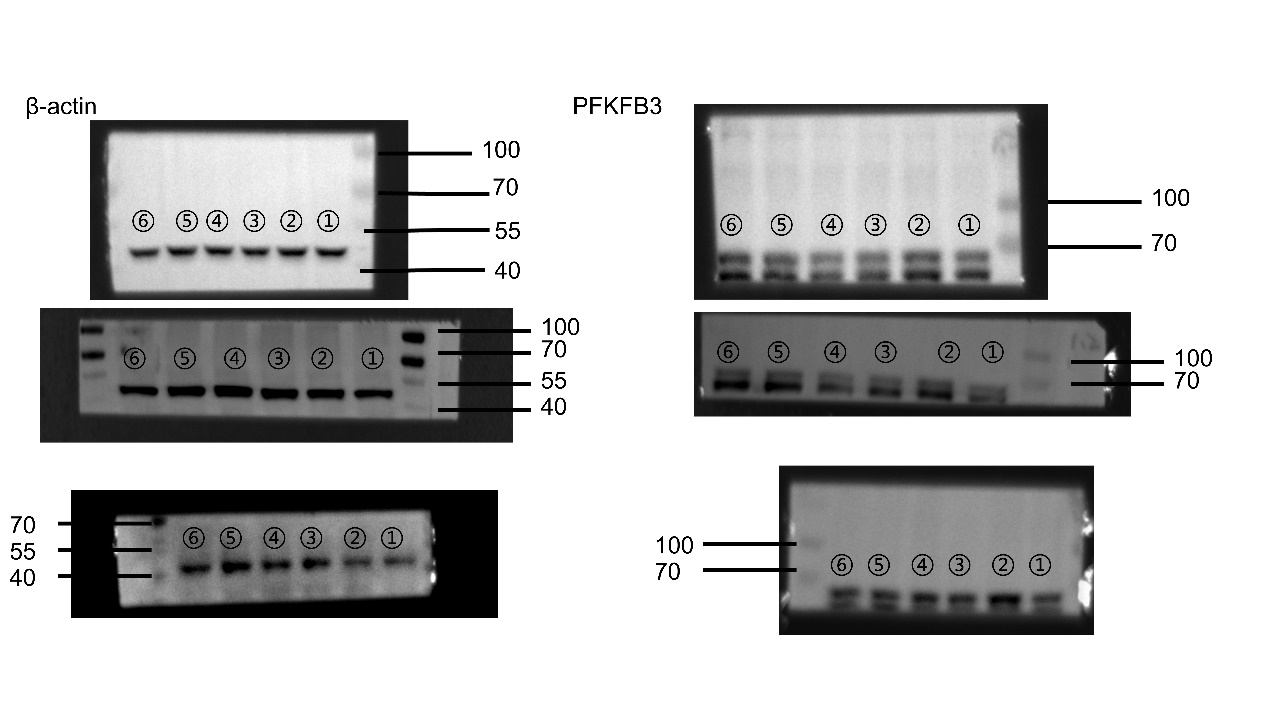


**Figure S21**. The original Western Blot graphs of β-actin and PFKFB3


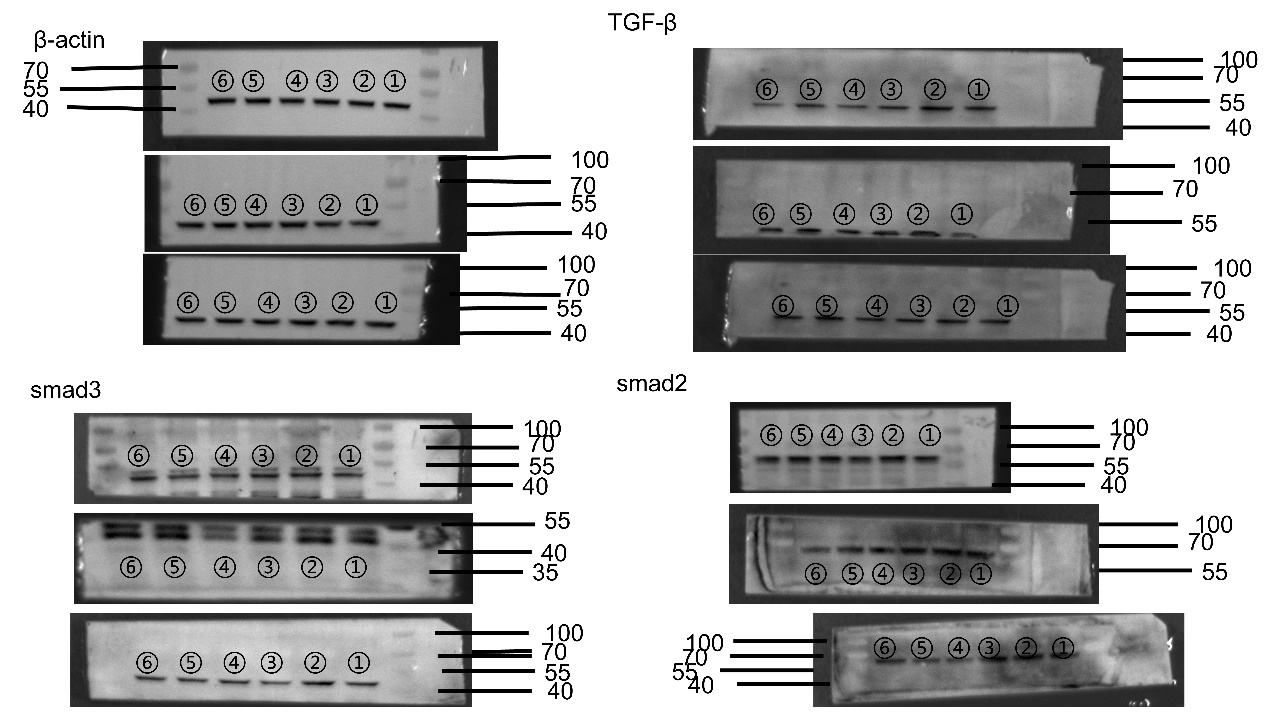


**Figure S22**. The original Western Blot graphs of β-actin, TGF-β, smad3, and smad2.

Table S1. The detailed information of primary and secondary antibodies used in this study.

| Target Antigen | Host Species | Clonality | Sequence | Catalog Number | Supplier | Dilution | RRID |
| --- | --- | --- | --- | --- | --- | --- | --- |
| PFKFB3 | Rabbit | Polyclonal | MPLELTQSRVQKIWVPVDHRPSLPRSCGPKLTNSPTVIVMVGLPARGKTYISKKLTRYLNWIGVPTKVFNVGEYRREAVKQYSSYNFFRPDNEEAMKVRKQCALAALRDVKSYLAKEGGQIAVFDATNTTRERRHMILHFAKENDFKAFFIESVCDDPTVVASNIMEVKISSPDYKDCNSAEAMDDFMKRISCYEASYQPLDPDKCDRDLSLIKVIDVGRRFLVNRVQDHIQSRIVYYLMNIHVQPRTIYLCRHGENEHNLQGRIGGDSGLSSRGKKFASALSKFVEEQNLKDLRVWTSQLKSTIQTAEALRLPYEQWKALNEIDAGVCEELTYEEIRDTYPEEYALREQDKYYYRYPTGESYQDLVQRLEPVIMELERQENVLVICHQAVLRCLLAYFLDKSAEEMPYLKCPLHTVLKLTPVAYGCRVESIYLNVESVCTHRERSEDAKKGPNPLMRRNSVTPLASPEPTKKPRINSFEEHVASTSAALPSCLPPEVPTQLPGQNMKGSRSSADSSRKH | A22317 | ABclonal | 1:1000 | AB_2844821 |
| TGF-β | Rabbit | Polyclonal | MPPSGLRLLLLLLPLLWLLVLTPGRPAAGLSTCKTIDMELVKRKRIEAIRGQILSKLRLASPPSQGEVPPGPLPEAVLALYNSTRDRVAGESAEPEPEPEADYYAKEVTRVLMVETHNEIYDKFKQSTHSIYMFFNTSELREAVPEPVLLSRAELRLLRLKLKVEQHVELYQKYSNNSWRYLSNRLLAPSDSPEWLSFDVTGVVRQWLSRGGEIEGFRLSAHCSCDSRDNTLQVDINGFTTGRRGDLATIHGMNRPFLLLMATPLERAQHLQSSRHRRALDTNYCFSSTEKNCCVRQLYIDFRKDLGWKWIHEPKGYHANFCLGPCPYIWSLDTQYSKVLALYNQHNPGASAAPCCVPQALEPLPIVYYVGRKPKVEQLSNMIVRSCKCS | AF1027 | Affinity | 1:1000 | AB_2835389 |
| Smad2 | Rabbit | Polyclonal | MSSILPFTPPVVKRLLGWKKSAGGSGGAGGGEQNGQEEKWCEKAVKSLVKKLKKTGRLDELEKAITTQNCNTKCVTIPSTCSEIWGLSTPNTIDQWDTTGLYSFSEQTRSLDGRLQVSHRKGLPHVIYCRLWRWPDLHSHHELKAIENCEYAFNLKKDEVCVNPYHYQRVETPVLPPVLVPRHTEILTELPPLDDYTHSIPENTNFPAGIEPQSNYIPETPPPGYISEDGETSDQQLNQSMDTGSPAELSPTTLSPVNHSLDLQPVTYSEPAFWCSIAYYELNQRVGETFHASQPSLTVDGFTDPSNSERFCLGLLSNVNRNATVEMTRRHIGRGVRLYYIGGEVFAECLSDSAI | 12570-1-AP | Proteintech | 1:5000 | AB_2193037 |
| Smad3 | Rabbit | Monoclonal | fusion protein | 12570-1-AP | Proteintech | 1:6000 | AB_2881879 |
| β-actin | Mouse | Monoclonal | MDDDIAALVVDNGSGMCKAGFAGDDAPRAVFPSIVGRPRHQGVMVGMGQKDSYVGDEAQSKRGILTLKYPIEHGIVTNWDDMEKIWHHTFYNELRVAPEEHPVLLTEAPLNPKANREKMTQIMFETFNTPAMYVAIQAVLSLYASGRTTGIVMDSGDGVTHTVPIYEGYALPHAILRLDLAGRDLTDYLMKILTERGYSFTTTAEREIVRDIKEKLCYVALDFEQEMATAASSSSLEKSYELPDGQVITIGNERFRCPEALFQPSFLGMESCGIHETTFNSIMKCDVDIRKDLYANTVLSGGTTMYPGIADRMQKEITALAPSTMKIKIIAPPERKYSVWIGGSILASLSTFQQMWISKQEYDESGPSIVHRKCF | AF7018 | Affinity | 1:5000 | AB_2839420 |
| Anti-Rabbit IgG | Goat | Polyclonal | -- |  | Zhongshan Jinqiao Biotechnology | 1:5000 | AB_2839429 |
| Anti-Mouse IgG | Goat | Polyclonal | -- |  | Zhongshan Jinqiao Biotechnology | 1:5000 | AB_2839430 |
